# Assessing the efficacy of coproduction to better understand the barriers to achieving sustainability in NHS chronic kidney services and create alternate pathways

**DOI:** 10.1111/hex.13391

**Published:** 2021-12-28

**Authors:** Leah Mc Laughlin, Gail Williams, Gareth Roberts, David Dallimore, David Fellowes, Joanne Popham, Joanna Charles, James Chess, Sarah Hirst Williams, Jonathan Mathews, Teri Howells, Judith Stone, Linzi Isaac, Jane Noyes

**Affiliations:** ^1^ School of Medical and Health Sciences Bangor University Bangor Wales UK; ^2^ Welsh Renal Clinical Network Welsh Health Specialised Services Committee Pontypridd Wales UK; ^3^ Cardiff and Vale University Health Board Cardiff Wales UK; ^4^ Patient Representative Swansea Wales UK; ^5^ Paul Popham Fund Renal Support Wales Swansea Wales UK; ^6^ Centre for Health Economics and Medicines Evaluation Bangor University, School of Medical and Health Sciences Bangor Wales UK; ^7^ Swansea Bay University Health Board Swansea Wales UK; ^8^ Betsi Cadwaladr University Health Board Bangor Wales UK; ^9^ Kidney Wales Foundation Cardiff Wales UK; ^10^ Kidney Care UK Alton UK

**Keywords:** coproduction, dialysis, family, kidney disease, patient, service improvement study, sustainability

## Abstract

**Context:**

Too many people living with chronic kidney disease are opting for and starting on hospital‐based dialysis compared to a home‐based kidney replacement therapy. Dialysis services are becoming financially unsustainable.

**Objective:**

This study aimed to assess the efficacy of coproductive research in chronic kidney disease service improvement to achieve greater sustainability.

**Design:**

A 2‐year coproductive service improvement study was conducted with multiple stakeholders with the specific intention of maximizing engagement with the national health kidney services, patients and public.

**Setting and Participants:**

A national health kidney service (3 health boards, 18 dialysis units), patients and families (*n* = 50), multidisciplinary teams including doctors, nurses, psychologists, social workers, and so forth (*n* = 68), kidney charities, independent dialysis service providers and wider social services were part of this study.

**Findings:**

Coproductive research identified underutilized resources (e.g., patients on home dialysis and social services) and their potential, highlighted unmet social care needs for patients and families and informed service redesign. Education packages were reimagined to support the home dialysis agenda including opportunities for wider service input. The impacts of one size fits all approaches to dialysis on specialist workforce skills were made clearer and also professional, patient and public perceptions of key sustainability policies.

**Discussion and Conclusions:**

Patient and key stakeholders mapped out new ways to link services to create more sustainable models of kidney health and social care. Maintaining principles of knowledge coproduction could help achieve financial sustainability and move towards more prudent adult chronic kidney disease services.

**Patient or Public Contribution:**

Involved in developing research questions, study design, management and conduct, interpretation of evidence and dissemination.

**Abbreviations**
WRCN: Welsh Renal Clinical Network
MDTs: Multi Disciplinary Teams
UHD: Unit Haemodialysis also referred to as hospital‐based dialysis
NICE: National Institute for Health and Care Excellence
PD: Peritoneal Dialysis
HHD: Home Haemodialysis
KRT: Kidney Replacement Therapy
BAME: Black, Asian, Minority, Ethnic
WKRU: Wales Kidney Research Unit

## 1 BACKGROUND

At least 10% of the global population is estimated to have chronic kidney disease (CKD).^1^ Kidney disease has a major impact on global health both in terms of mortality and disease burden, with these numbers increasing year on year.^2^, ^3^, ^4^, ^5^ In 2010, more than 2.6 million people received a kidney replacement treatment (KRT—the collective name for either a transplant or dialysis), and numbers are projected to more than double by 2030, with the biggest increase coming from low‐ and middle‐income countries.^6^ People who have developed kidney failure very often have multiple comorbidities,^7^ are older, more frail, more deprived^8^, ^9^ and disease progression is not easily predicted.^10^ They can rely on multiple health and social care services for their care and support.^11^ While KRTs have been available for decades in high‐income countries, overall, little is known about the optimal way to coordinate, finance and regulate people with CKD from diagnosis, access to KRT and their overall care and support.^12^

There are three main options available when a person goes into kidney failure: transplant, dialysis and supportive care without dialysis (sometimes called conservative management). Dialysis can occur in a hospital setting called unit haemodialysis (UHD) undertaken three times a week for 4 hour sessions at a time, or at home. If a person chooses home dialysis, there are generally two types of dialysis (peritoneal dialysis and home haemodialysis) available (depending on clinical suitability), both of which can be administered during the day or overnight depending on people's preferences and outcomes. National Institute for Health and Care Excellence (NICE) guidelines estimate that a minimum of 30% of the current dialysis population in the United Kingdom could be on home dialysis. NICE guidance also acknowledges the substantial impact that the different treatments will have on lifestyle and that people's values and preferences must be taken into account when presenting KRT options to them and their family.^13^ Further background to the development of home therapies and global trends is presented in File S1.

### 1.1 Sustainability and the Welsh National Health Service context

Wales is one of the four devolved nations of the United Kingdom, with a devolved healthcare system and a population of around 3 million. The incidence and prevalence of CKD are higher in Wales than in the rest of the United Kingdom, affecting 6%–8% of the Welsh population (around 200,000 people).^14^ More than 3000 people are currently on KRT in Wales, with this number increasing year on year.^15^ In 2017, Wales had higher numbers of people start on home therapies and higher numbers of people currently on home therapies compared to the UK average (Box 1). For the first time in 10 years, commissioners of kidney services in Wales (Welsh Renal Clinical Network) were forced to request a net increase in investment from NHS Wales to sustain the dialysis service for increasing numbers of people. This exponential increase is considered unsustainable as money is finite and too many people are unnecessarily opting for more expensive UHD.

BOX 1 **Summary percentages of the Welsh population starting and currently on dialysis compared to the UK average 2017^16^**

1

**Table:** 

	**Wales**	**UK**
Percentage of people to start on home therapies	23%	21.6%
Percentage of prevalent dialysis patients on home dialysis	20.3%	16.8%

The National Health Service (NHS) adopts the well‐established ‘Three Pillars of Sustainability: Social, Economic and Environmental’ model across all its health service improvement strategies and agendas for change.^17^ In Wales, the Wellbeing of Future Generations (Wales) Act 2015^18^ and the Social Services and Wellbeing (Wales) Act 2014^19^ provide the key policy contexts for all health and social care including three pillar plans for more sustainable services. The underpinning healthcare policy in Wales is Prudent Healthcare,^20^ which specifically recognizes the interdependence of specific challenges in creating more sustainable health and social care services. Examples of prudent healthcare plans include ‘A Healthier Wales’^21^ which puts care and support at home at the heart of service improvement developments,^22^ long‐term ambitions for health and social care systems to work together and, where possible, to shift services out of hospitals into the communities.^23^

A key principle of prudent healthcare is coproduction, defined as *‘a way of working whereby citizens and decision makers, or people who use services, family, carers and service providers work together to create a decision or service which works for them all’.* ^24^ Since the implementation of prudent healthcare in 2014, Welsh policy makers encourage coproduction as the default way of working and are increasingly asking that the evidence which informs decision making is coproduced.^19^, ^25^, ^26^ Increasing the number of people on home dialysis in Wales is a prudent healthcare policy.^20^

### 1.2 Coproduction in sustainable health services contexts

In a research context, coproduction is broadly defined as ‘*an approach in which researchers, practitioners and the public work together, sharing power and responsibility from the start to the end of the project, including the generation of knowledge’.* ^27^ Coproduction is becoming more common, and new models of coproduction and methods of assessment are constantly being developed.^28^, ^29^, ^30^ Recent coproductive health research reports on the capacity of coproduction to increase impact, facilitate knowledge translation, identify underused or unrecognized resources (people, services, networks), improve information and education processes by tailoring to individual need and support overall health improvement initiatives.^31^, ^32^, ^33^, ^34^ Increases in the breadth of examples of coproductive health services research have also highlighted challenges to coproduction. These include costs, resources, training, time, cultural differences and misunderstandings of what is (and is not) coproductive research.^35^, ^36^, ^37^, ^38^, ^39^, ^40^, ^41^ Increasingly, however, health research is turning to the global contexts of coproduction, recognizing the potential value (in health service and policy contexts) while at the same time addressing known barriers and new challenges.^33^, ^34^, ^36^, ^42^, ^43^, ^44^

Previous attempts to address low uptake of home dialysis have centred around shared decision making (SDM), specifically, the Making Good Decisions In Collaboration (MAGIC) model developed for the UK NHS.^45^ MAGIC provides a template based on ‘choice, option, decision talk’ to help professionals implement SDM in clinical settings and explains the patient's experience as a journey from uninformed to informed through building rapport, mutual respect and active listening. SDM is widely recognized as best practice, and yet has been shown to be problematic to implement across the relevant healthcare settings as the necessary systems, infrastructures and wider support networks (e.g., well designed and validated decision support aids, patient empowerment, clinician training, culture shifts and system bureaucracies) are either not available or not working in ways that promote SDM models of care. SDM is also a key principle of prudent healthcare that has been implemented across international health contexts and policies, for example, the ‘1000 Lives Improvement’ programme,^47^ and changing the law in consent for organ donation.^48^

## 2 METHODS

### 2.1 Study context

**Figure 1:**
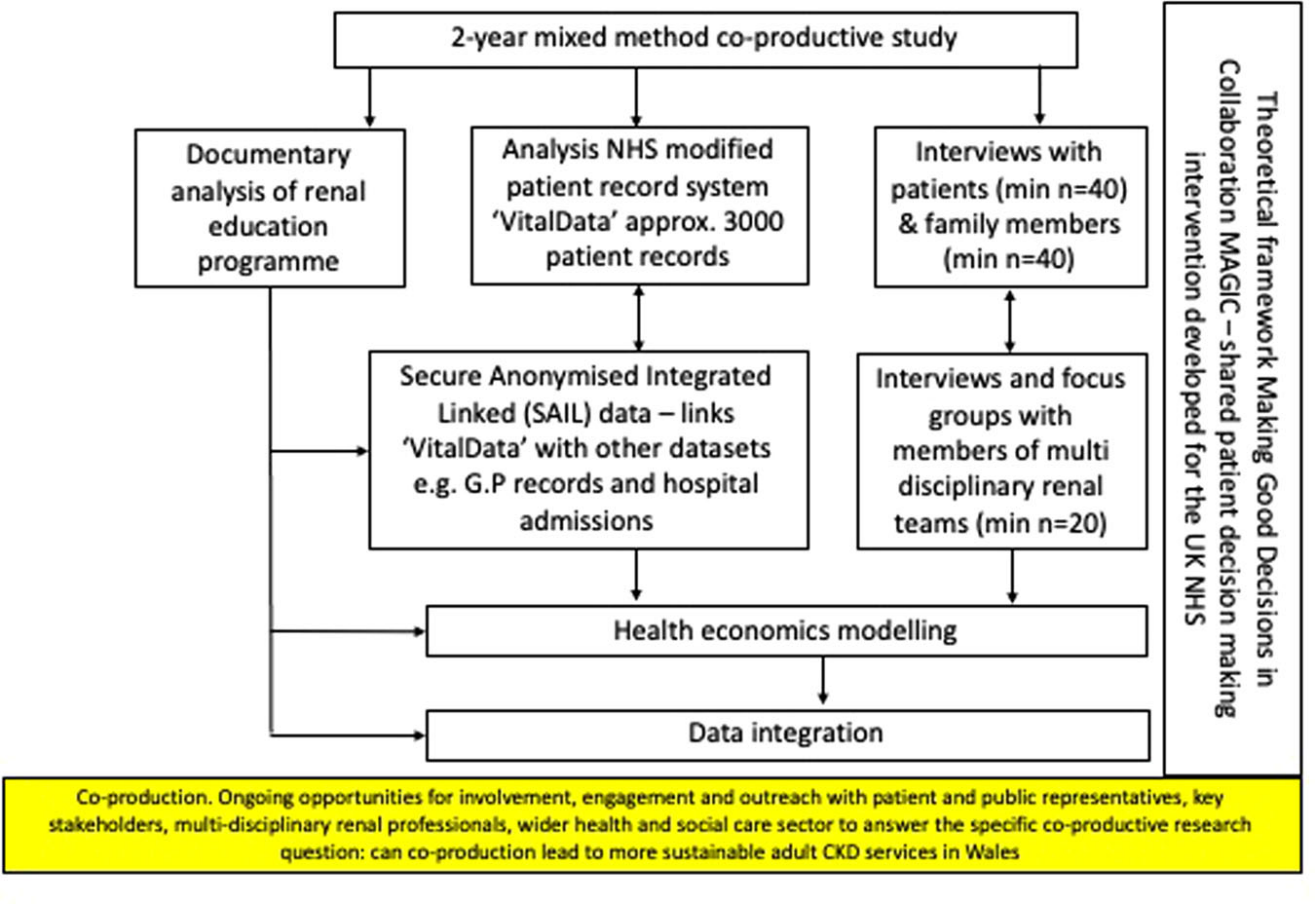
Overall study diagram, highlighting the coproductive elements and the focus of this paper

We conducted a study with two elements—a health service improvement element and an empirical element that included an analysis of epidemiological big data, costs of dialysis modalities and a qualitative study of patient and carer perspectives. In this paper, we specifically focus on the health service improvement element and the coproductive methods and outcomes, which were deployed to help answer a specific coproductive research question: Can coproduction lead to more sustainable adult CKD services in Wales? This question was predefined by the funder as part of a themed national health and social care funding call under the umbrella of research for the patient and public benefit scheme. The question was interpreted for this study as ‘assessing the efficacy of coproduction to better understand the barriers to home dialysis and map alternate pathways’. Full details of the overall study are available in the published protocol.^49^ We summarize the overall study in Figure 1 and at the same time highlight the specific health service improvement and coproduction elements that this paper focusses on. The objective of this study was to ‘assess the efficacy of co‐productive research in chronic kidney disease service improvement to achieve greater sustainability’. The core research team were multidisciplinary and included academics from health services and systems research, health economists, the lead kidney nurse for Wales, kidney service commissioners, nephrologists and people living with kidney disease.

### 2.2 Settings and participants

**Figure 2:**
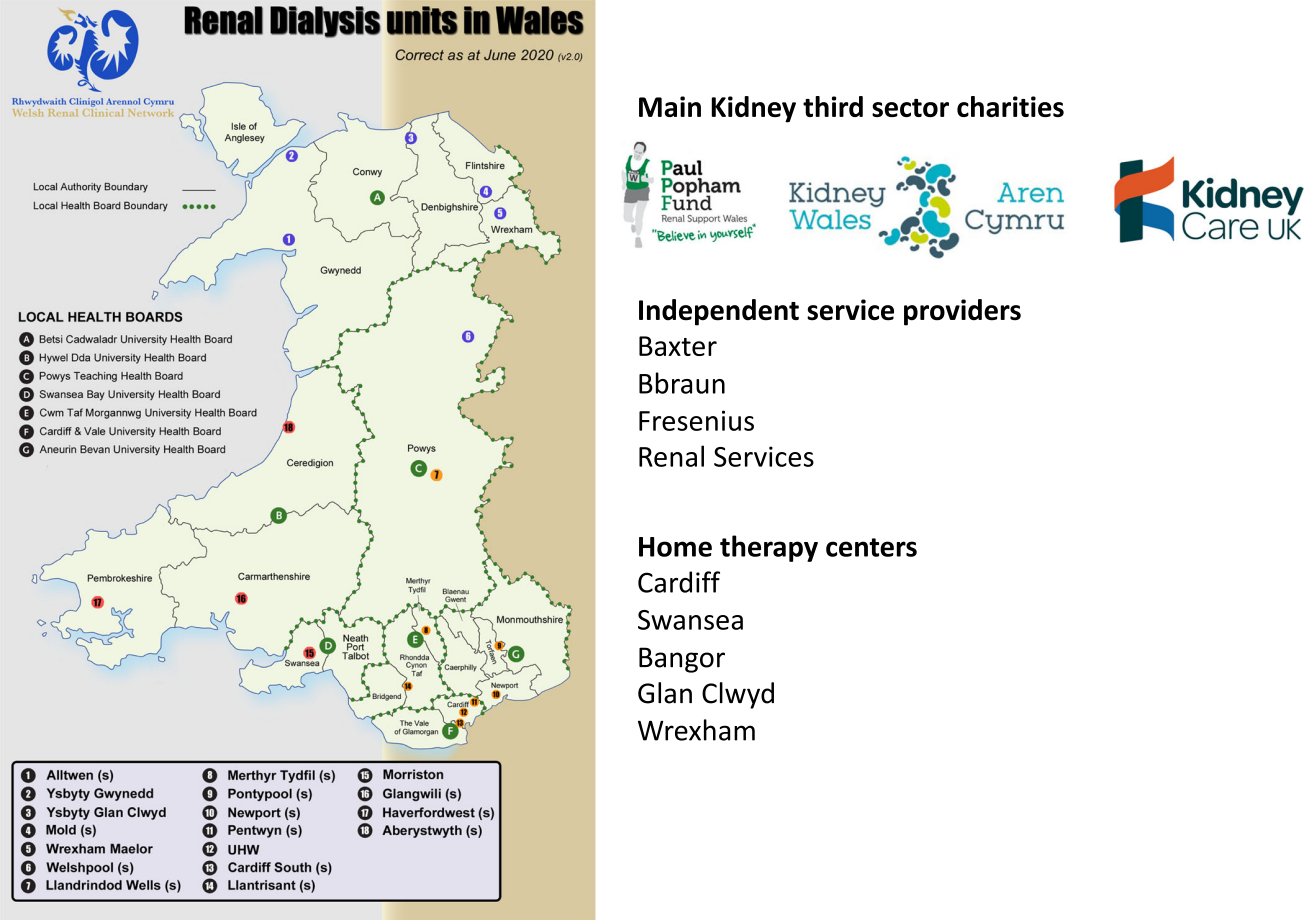
Map of kidney services across Wales

This was a 2‐year coproductive study (October 18–20). The following were either key partners or settings in the study: kidney services covering all kidney healthcare in Wales (Figure 2), commissioners of welsh kidney services—Welsh Renal Clinical Network (WRCN), kidney third sector services (Paul Popham Fund, Kidney Care UK, Kidney Wales), wider third sector services (e.g., Carers Wales, Citizens Advice, Care and Repair Cymru, Action for Elders), local councils and local authorities in Wales, industry (independent dialysis service providers Baxter, B. Braun, Fresenius), social service commissioners in Wales, people living with kidney disease, their family members and close friends. Overall numbers of participants from the all‐Wales NHS kidney team workforce are summarized in Box 2. As a coproductive study, all people living with kidney disease in Wales and their families were eligible for inclusion in the study. We used a purposive sampling frame to achieve a maximum‐variation sample. People were invited by healthcare professionals and kidney charities to participate in the various coproductive elements of the study. Healthcare professionals included those most directly involved in dialysis decision making and care (e.g., specialist nurses and nephrologists) and included the wider multidisciplinary team to ensure that the whole kidney service was represented.

BOX 2 **Overall numbers of NHS kidney multidisciplinary teams, patients and family members who contributed to the coproduction**

1

**Table:** 

Clinical consultants	*n* = 12
Nurses and nurse managers, including predialysis education specialists, home therapies specialists and transplant specialists	*n* = 30
Allied health professionals, including psychologists, dieticians, occupational therapists, physiotherapists, pharmacists	*n* = 20
Specialist renal social workers, counsellors and additional specialist services, for example, youth workers	*n* = 6
People living with kidney disease including family members from across Wales on various kidney replacement therapies and people who had yet to start dialysis	*n* = 50

### 2.3 Coproduction

Specific coproduction principles that focussed on sustainability were adapted from Norstrom et al.,^50^ who produced a set of four general principles to underpin high‐quality knowledge coproduction for sustainability research. These principles were then used to map the coproduction processes throughout the study and acted as the theoretical framework (Box 3). The broad aim was to assess the efficacy of coproductive research in a health service improvement study. In this context, that translated to better understanding of the barriers to home dialysis and mapping of alternate pathways. The goal was to cocreate a new vision for home dialysis services and achieve greater sustainability.

BOX 3. **Principles for knowledge coproduction in sustainability research ^50^**

1

- *Context‐based*: The process should be grounded in an understanding of how a challenge emerged, how it is affected by its particular social, economic, political and ecological contexts and the different beliefs and needs of those affected by it.
- *Pluralistic*: The process should explicitly recognize a range of perspectives, knowledge and expertise and consider gender, ethnicity and age in development.
- *Goal‐oriented*: The process should articulate clearly defined, shared and meaningful goals that are related to the challenge at hand.
- *Interactive*: The process should allow for ongoing learning among actors, active engagement and frequent interactions.

We also embedded the six UK standards for public involvement into the study (inclusive opportunities, working together, support and learning, governance, communications and impact) to ensure that coproduction was at the centre of all research activities and processes throughout. The UK standards are mapped against the full range of coproductive activities in this study with examples in File S2. All of the coproductive patient and public involvement was conceptualized as a partnership. Coproductive partners were coproducing and interpreting the research together and as such were fully informed of their roles and expectations in invitations. No formal consent procedures were required as they were not research participants.

We present the purpose of coproduction mapped onto the principles of knowledge coproduction in sustainability research in Table 1. Table 1 also includes an ‘interactive co‐production activity log’ with details of the meetings, events and engagements including numbers who attended with a breakdown of kidney professionals, the public and people living with kidney disease over the 2‐year timeframe of the study. We offered travel and any out‐of‐pocket expenses for people with kidney disease and the public to attend any coproductive events or meetings organized by the research team. Coapplicants who were people with kidney disease were paid a rate in line with the national standards for PPI throughout the study.^51^

**Table 1:** Principles of knowledge coproduction mapped against coproductive elements and clarifying their purpose

Principles of knowledge coproduction mapped against key components of the study, of which coproduction was essential						
		Purpose	Context‐based methods	Pluralistic methods	Goal‐orientated methods	Interactive^a^ (see coproduction interaction log below for further details)
1	Creating a vision of a more sustainable adult kidney service in Wales	There were two main purposes to this activity: (1) To unpick the key elements of the current renal service that were unsustainable from the perspective of coproductive partners and (2) highlight some of the ways in which existing services and resources could be reconfigured or redesigned to support a future sustainable renal service	Academic members of the research team with PPI input produced a framework with the headings; ‘what is currently unsustainable in adult kidney services’, ‘what does good look like’, ‘how can coproduction help’ and ‘what is difficult to achieve through co‐production’. The headings were then linked to the various perspectives: people living with kidney disease; family members; multi‐disciplinary health and social care professionals; NHS; industry; government; third sector; and wider social contexts (Table 2)	The table was then shared with all the multiple key stakeholders for their input. This included NHS specialist; nephrologists, transplant physicians, social workers, physiotherapists, dieticians, clinical psychologists, counsellors, youth workers and specialist nurses; commissioners; Industry and independent service providers; renal third sector service providers; wider third sector services; social service commissioners; people living with kidney disease; family members and close friends; and the public	The table presented current challenges and potential consequences in tandem with the vision for what good looks like; this included immediate challenges to the Welsh renal service such as overspending, as well as wider challenges outside of NHS secondary renal care, such as overall population health and deprivation. The template presented problems alongside their ‘ideal’ solution and how the multiple audiences could help achieve the solution, in particular, how people living with kidney disease could influence change. As the table grew, we extracted elements that were more relevant to specific groups (e.g., social services) and presented specific problems, ideal solutions and novel ideas for coproduction back to the stakeholders who would be most affected by any changes	We shared the list at specifically curated coproductive meetings with the multidisciplinary teams in North and South Wales, which included patients and family members; operational engagements led by the NHS such as quarterly all Wales Specialist Nurse meetings; and Renal third sector partner events such as transplant cafes. We also shared via small group face‐to‐face meetings with specific members of the MDTs, visits to dialysis units and centres, non‐kidney‐related events (e.g., the festival of social sciences in North Wales and research festivals in Wales National Museum). We shared via email, telephone and face‐to‐face correspondence with patients and later over zoom meetings and curated webinars following the COVID‐19 outbreak. People inputted directly into the table by writing into it, group and individual face‐to‐face feedback, emails and by reviewing the materials directly with academic members of the research team. People often fed back informally following presentations and events throughout the study. We used post it notes, traffic light systems (green = good, amber = don't know/irrelevant, red = bad) and paper cut outs to facilitate the mappings
2	Coproductive redesigning of service pathways in adult chronic kidney disease	The purpose of this coproductive activity was to gain an understanding of the current renal service pathways, how they connect and interact and to design new pathways that could potentially address the sustainability issues as seen from the multiple perspectives. This particular component was deemed essential by key stakeholders (especially commissioners) to address the overall research question, set by a research funding scheme designed to focus on health service improvement and demonstrate impact in the short to medium term	The original draft of the service improvement pathway/‘pathways to home’ were designed by the lead renal nurse for Wales, also a study coapplicant who had extensive knowledge of the current state of Welsh renal services and were very knowledgeable about wider UK and global contexts and service improvement initiatives. The initial drafting also had patient input (Figure 3)	The pathways were shared with the NHS multidisciplinary teams, in particular, the specialist renal nurses in predialysis and home therapies; professionals allied to medicine including physiotherapists, dieticians and clinical psychologists; renal social workers and youth workers; and people living with kidney disease, family members, renal third sector CEOs and staff working for renal charities and volunteer peer‐to‐peer support workers—many of whom were either living with kidney disease or a family member of someone with kidney disease	Sharing included a preamble, with the current service pathway as a backdrop. This helped to highlight gaps in terms of what the service did not currently have, and then move onto potentials for improvement across the multiple service pathways. The before and after snap shots helped set out the vision for what renal services could look like at a fairly high level across Wales and to more easily present this in relevant ways to the multiple stakeholders for their input	Academic members of the research team attended quarterly NHS specialist nurse meetings, quarterly professionals allied to medicine meetings (which were also attended by patients and renal third sector partners) and worked with renal charity partners to set up specially coordinated zoom meetings to present to people living with kidney disease, family members and charity provider executives. Webinars were produced and made available online and promoted on social media for people to refer and feedback into via comments, telephone, zoom and face‐to‐face meetings
3	Coproductive Review of education materials and processes	The purpose was to gain a better understanding of the predialysis education programme across Wales, how it was delivered, by whom and to better understand any variations in practice and uptake of home dialysis	Specialist predialysis nurses are primarily responsible for explaining the available treatment options (using various education materials) to people who will need a renal replacement therapy and their family. They also support people while transitioning to starting dialysis or moving to a different treatment and managing symptoms with conservative care and liaise with the multidisciplinary teams (including consultants) about patient care. There are around 15 working across five renal centres in Wales. Despite education processes looking broadly similar across centres on paper, reviews of the number of people on home dialysis varied significantly: only one centre was achieving the national guidelines of 30% on home dialysis	The research team invited the lead nurse to come and present at a specially coordinated coproductive multidisciplinary meeting (with patients and family members in their service also attending) and explain how their service worked and what they felt they were doing differently to everybody else. Academic members of the research team were also invited (following a study update at an NHS quarterly meeting) to attend a house visit with a specialist nurse (from a different centre) to observe their initial conversations with a person needing RRT and their family members	Early on, we explained the aims of the study and highlighted the crucial role that the predialysis specialist nurses played in the research and their role in supporting patients. In this instance, the research team were especially keen to find out more about the potential impacts that the different service configurations had on the uptake of home dialysis, as well as how nurses were delivering education in terms of their priorities and practices	The lead nurse presentation was followed by a question and answer session to an MDT room of practitioners from different services, patients and family members. Having patients and family members attend who were under that particular service care also enabled them to talk about their recent experiences and views on the barriers to home therapies from their perspectives. The field visit to the predialysis persons' home with their family was in itself highly interactive—academic members of the research team and the predialysis nurse were able to converse in detail informally about their education processes and practices as a whole and with that particular family following the consultation
5	Coproductive review of patient and public attitudes to costs	The purpose was to learn more about public attitudes to the costs of renal healthcare and services to better understand if and how costs are considered in patient and clinical decision making and how the conceptualization of costs could be framed and apportioned to health and social services in a sustainability context looking forward	Nobody knew the actual costs of RRTs in Wales. Previous estimates were outdated and likely to be inaccurate. Wider literature provided little up‐to‐date insights in terms of NHS spend, especially in home haemodialysis. Hidden costs for example job loss, part‐time working, childcare, housing modifications and family impacts such as switching to full‐time carers were scarcely reported. There were various perspectives on costs; however, it was widely assumed within the NHS that hospital or unit dialysis was the most expensive but we knew little about public understandings, attitudes and perceptions of costs of dialysis	Academic members of the research team approached patients and the public to ask about their views on dialysis costs, potential savings and perceptions of shifting services between health and social care. We also approached social services commissioners in Wales to learn from their perspectives	The costs of dialysis services are increasing globally. The coproduction aimed to learn about views and perceptions of the costs of different RRTs in Wales, and specifically how people felt about suggested models for cost savings from the multiple stakeholder perspectives	Academic members of the research team attended several nonkidney disease‐related public events such as hosting a market stall at St. David's Day parade, presenting at All Wales involving people festival and attending local community group meetings with BAME individuals and groups to enhance opportunities for learning and interactive feedback with the public in Wales. We also attended the National Transplant games in Newport and the Welsh Renal Clinical Networks Renal Roadshow with a banner and preamble of the most recent evidence of costs of dialysis. We delivered presentations at kidney patient‐led conferences and meetings to discuss costs and gather perspectives, with larger and smaller groups of kidney patients over 18 months

Coproduction log							
PPI and professional outreach and engagement dialysis options and choices							
Event	Type of activity	Type of PPI	Date	Total number of attendees	Total number of renal profs	Number of public members	Number of patients
Give the name of the event, in brief	Please specify the main type of activity	Please specify if: involvement, engagement outreach	Please select from the dropdown menu	Give the total number of attendees	Give the total number of attendees	Give the number of lay/public members attending event	Give the number of patients attending the event
Presentation to CNS nurses CKD meeting, llandrinod wells, 26.11.18	Presentation	Involvement	26.11.18	15	14	1	1
Presentation to home therapies nurses, llandrinod, 07.12.18	Presentation	Involvement	07.12.18	12	11	3	3
Presentation to BAME group Wyndam Day Centre Riverside, 30.11.18	Presentation	Engagement	30.11.18	12	0	12	12
Presentation to patient focus group, Cardiff WKRU 06.11.18	Presentation	Involvement	06.11.18	7	3	1	3
Stand HCRW AGM all	Stand	Outreach	25.10.18	100	8	10	2
Presentation at WKRU AGM Swansea 28.11.18	Presentation	Engagement	28.11.18	50	38	12	10
Attendance. Community‐powered health supporting better outcomes for all ages	Attendance	Engagement	23.10.18	25	2	15	5
Attendance Castle Bingo Newport Rd	Attendance	Engagement	06.11.18	5	0	5	5
Presentation National Home Therapies Group	Presentation	Involvement	07.12.18	20	17	2	2
Presentation kidney clinical community England	Presentation	Engagement	10.12.18	12	10	2	1
Presentation Clinicians Morriston internal meeting group	Presentation	Engagement	20.12.18	7	7	0	0
Presentation ABMU CNSs Morriston	Presentation	Engagement	08.01.19	4	4	0	0
Presentation C&V CNSs C&V	Presentation	Involvement	16.01.19	5	5		
Presentation Renal Business meeting Morristion	Presentation	Engagement	22.01.19	19	19		
Update Presentation National Home Therapies Road show	Talk	Engagement	25.01.19	12	12		
Meeting Race Council Cymru	Meeting	Outreach	29.01.19	1		1	
Presentation to CNS nurses North Wales	Presentation	Engagement	05.02.19	6	6		
Catch up C&V CNSs Nurses	Meeting	Involvement	14.01.19	5	5		
Glan Clwyd Renal Patient Support Group	Presentation	Engagement	#########	8	1	7	7
Meeting Swansea CNSs/shared care nurses	Meeting	Involvement	19.02.19	3	3		
Meeting lead Renal Nurse	Meeting	Involvement	22.02.19	1	1		
Reference Group meeting	Meeting	Involvement	#########	14	5	1	1
Glan Clwyd Renal Unit	Meeting	Coproduction	#########	4		0	4
St David's Day Parade, St Davids Pembrokeshire	Stand	Involvement/outreach	3.0319	40	0	40	5
Ysbyty Gwynedd Renal Unit	Meeting	Coproduction	#########	3	3	0	0
Attendance 'co‐producing research' how do we share power NIHR	Attendance	Engagement	12.03.19	70	0	30	10
Public Health Wales 2019 conference	Poster Presentation	Engagement	13.03.19	100	0	20	10
National Home Therapies Group	Meeting	Involvement	29.04.19	20	18	2	2
CNO conference presentation	Presentation	Engagement	08.05.19	20	2	2	2
Dialysis Options and Choices Key stakeholder meeting	Meeting	Involvement	15.05.19	20	12	2	6
Paul Popham Conference presentation and stand	Presentation/stand	Involvement/engagement	17.05.19	40	20	5	20
CHEME Bangor University Invited Seminar	Presentation	Engagement	14.05.19	15	0	15	1
Meeting Kidney Research UK	Meeting	Engagement	26.06.19	4	0	2	1
Renal Roadshow Morriston	Stand	Involvement	04.07.19	40	20	10	10
National Home Therapies Meeting	meeting	Involvement	12.07.19	30	27	2	1
HCRW Lets Talk Research Annual Involving People Conference	Presentation	Engagement	27.07.19	50	50	50	2
Transplant Games Newport	Stand	Outreach	28.07.19	100	0	100	50
Meeting with‐ Lead Nurse/Directorate manager	Meeting	Involvement	12.08.19	1	1		
Meeting‐ Kidney Wales patient advocate	Meeting	Involvement	15.08.19	1	1		
Visit Carmarthen Dialysis Unit	Stand	Engagement	29.08.19	7			7
Visit Llandrindod Dialysis Unit	Stand	Engagement	30.08.19	6			6
Presentation and meeting with Paul Popham Befrienders	Meeting	Involvement	12.09.19	10		2	8
Third wider stakeholder meeting	Meeting	Involvement	18.09.19	30	18	8	4
Presentation National Home Therapies Group	Presentation	Involvement	20.09.19	15	12	1	3
HCRW annual conference—Collaboration and Partnerships	Presentation	Engagement	03.10.19	50+		50+	1 Kidney patient was a co presenter
National Audit Presentation			04.10.19				
Cardiff and Vale R&D conference presentation	Presentation	Engagement	15.10.19	40+		40+	
Attendance at National Health and Wellbeing Professionals Group	Meeting	Involvement	22.10.19	10			10
Presentation at Festival of Social Sciences 'Social Prescribing Network Event, Bangor, Bangor University	Presentation/stand	Involvement	07.11.19	25	0	23	1
Workshop with Paul Popham Befrienders, Swansea	Workshop	Involvement	12.11.19	9	0	1	8
Research findings mapping roadmaps	Meeting/presentation	Involvement	13.11.19	5	1		4
National CKD meeting	Meeting/presentation	Involvement	22.11.19	13	9	2	2
Coproduction meeting on home therapies	Meeting/presentation	Involvement	25.11.19	35	12	10	13
Field visit with CKD nurse to home	Field work	Involvement	27.11.19	1	1		
Conservative Management meeting ACP	Meeting	Engagement	28.11.19	8	6	2	
Transplant café Swansea	Meeting/presentation	Involvement	13.12.19	12	4	1	7
Patient meeting	Meeting	Involvement	13.01.20	2			1
Transplant café Cardiff	Meeting	Involvement	15.01.20	9	3	1	5
Renal peer review training	Meeting	Involvement	17.01.20	30	28	2	
Befriender steering committee	Meeting	Involvement	23.01.20	9	5	3	1
Home therapies café	Meeting	Involvement	07.02.20	9		6	3
National home therapies group	Meeting	Involvement	10.02.20	15	13	2	
National CKD meeting	Meeting	Involvement	21.02.20	12	10	2	
WKRU AGM	Meeting/presentation	Involvement	27.07.20	80	60	10	10
Video of patient presentation shown on YouTube over world kidney day describing his live donor journey	Video	Involvement	12.03.20	Over 100 views online			
							
Types of events (hosted/organized by Infrastructure group)							
Seminar							
Conference							
Workshop							
Open‐lab							
Open‐office							
Presentation							

Abbreviations: CNS, clinical nurse specialist; MDT, multidisciplinary team; RRT, renal replacement therapy; WKRU, Wales Kidney Research Unit.

^a^ We offered travel and any out‐of‐pocket expenses for patients and the public to attend any coproductive events or meetings organized by the research team. Coapplicants who were patients were paid a rate in line with the national standards for PPI throughout the study.^56^

There were four specific components of the health improvement study, of which coproduction was fundamental to address:

1. To create a vision of a more sustainable adult kidney service in Wales,
2. To redesign service pathways in adult CKD,
3. To review patient education materials and processes and
4. To better understand patient and public attitudes towards the costs of kidney care and services.

In the following section, we summarize how and what data were collected and analysed for each coproductive element, and how data were used to achieve the intended outcomes.

1. To create a vision of a more sustainable adult kidney service in Wales.

- Data collection
  The multidisciplinary research team, which included people with kidney disease, produced a framework with the headings ‘what is currently unsustainable in adult kidney services’, ‘what does good look like’, ‘how can co‐production help’ and ‘what is difficult to achieve through co‐production’. The headings were then linked to the various perspectives: people living with kidney disease, family members, multidisciplinary health and social care professionals, NHS, independent dialysis service providers, government, third sector and wider social contexts (Table 2).
  People with kidney disease worked with the research team to cocreate Table 2. They had input across every perspective by identifying sustainability issues and inputting into potential solutions through coproduction as well as helping produce subheadings to ensure that all elements of the wider services were included. To facilitate this, the research team arranged two specifically curated coproductive meetings with the multidisciplinary teams in North (25 November 2019) and South Wales (15 May 2019). We contacted the predialysis nurse specialists for each team and asked them to send out invites to people living with kidney disease and their families. We prepared a brief poster advertising the meeting and requested that people get in touch either by phone or email to book their places. We also invited nephrologists, predialysis nurse specialists and representatives from independent dialysis service providers (Baxter, B.braun, Fresenius) via email. Members of the research team are part of the Wales Kidney Research Unit, and these stakeholders were known to the academic team; those who were not immediately known were contacted by NHS members of the research team. Kidney charity representatives were also invited.
  To bolster these specifically curated meetings, the evolving framework in Table 2 was shared with stakeholders across Wales from November 2018 to March 2020 (16 months) to provide their input. Members of the research team attended regular meetings and events hosted by the coproductive partners, for example, operational engagements led by the NHS such as quarterly all‐Wales specialist nurse meetings and kidney third sector partner support events such as ‘transplant cafes’. Members of the research team who were patients also gathered anonymized information by speaking to patients in their roles as peer support workers and feeding information back to the research team throughout the data collection window.
- Data analysis

**List of key events and attendees**

**North Wales curated meeting:** Total *n* = 30, *n* = 18 renal professionals (nephrologists, kidney specialist nurses), *n* = 8 family members, *n* = 4 people with kidney disease

**South Wales curated meeting:** Total *n* = 20, *n* = 12 renal professionals (nephrologists, social workers, youth workers, specialist nurses), *n* = 2 family member, *n* = 2 people with kidney disease, *n* = 4 kidney industry partners.

**Transplant cafés**: Total *n* = 21, *n* = 7 renal professionals (specialist nurses, social workers clinical psychologists), *n* = 12 people with kidney disease, *n* = 2 industry partners.

**Table 2:** How can coproduction improve the sustainability of kidney services in Wales

	What is unsustainable (and barriers to sustainability) in the current adult CKD service in Wales	What does a sustainable kidney service look like for adult CKD services in Wales. ‘What does good look like?’	What can be changed through coproduction?
People living with kidney disease (PLKD)			
Decision making	∐ PLKD who struggle to make a decision/remain ‘undecided’ for longer tend to opt for unit haemodialysis (UHD). ∐ Some PLKD ‘bury their heads in the sand’, pretend it is not happening and delay making a decision. ∐ Unlike other diseases, CKD progression can be unpredictable. It can be a long‐term case of just ‘seeing how things go’; people avoid making decisions because of the unpredictability of progress. ∐ PLKD on certain pathways, for example, ‘pre‐emptive transplant pathway’ often become so fixated with not wanting dialysis that they refuse to plan for another pathway and will not engage with any dialysis conversations. ∐ Many PLKD tend to make a decision and then justify it retrospectively, rather than weighing up the pros and cons of each option. ∐ Often by the time patients come to make a decision, they have accumulated a mass of mis information.	∐ Every patient in Wales is supported to make an informed decision in a timely way based on clinical recommendations and PLKD values and preferences. ∐ Education is personalized and tailored to individual PLKD needs. ∐ Education provided, and decision made a minimum of 1 year before treatment is needed (2018 NICE guidelines). ∐ Dialysis options are considered alongside all transplant options. ∐ SDM is applied with a home‐first approach. Additional time and resources are applied where appropriate to unpick barriers to home therapies and work with PKLD and family to explore all home‐based dialysis options. ∐ Peer‐to‐peer support workers are introduced early in the processes and options for patients to talk to others is routinely offered.	∐ PLKD and peer support workers (who have been through these experiences) have the best potential to alleviate fears, encourage people to accept the disease prognosis and make decisions in a timely way. ∐ PLKD on home therapies can share their positive experiences of home therapies, their personal perspectives and work with health and social care teams to change to a home‐first approach.
Demographics	∐ PLKD from areas of deprivation/low socioeconomic status tend to choose UHD. ∐ Many PLKD from areas of deprivation/low socioeconomic status are more familiar with ‘being told’ what to do. ‘You are the doctor, you tell me what I should do’. They find it more difficult to practice SDM.	∐ PLKD from lower income/areas of deprivation are supported through health and social care services (e.g. welfare and benefits) for any additional (hidden) costs associated with home therapies. ∐ Additional Shared Decision Making (SDM) approaches are used to support people who struggle to assert their needs and values, as well as tailored peer‐to‐peer support.	∐ PLKD on home therapies and peer‐to‐peer support workers can support health and social care professionals to identify patients who are not currently on a home pathway, but would benefit from being on one.
Social contexts and services	∐ Many PLKD (in particular older) patients opt for UHD as they benefit from socializing with staff, other PLKD and ‘getting out of the house’. ∐ PLKD build relationships with staff in their clinics and form meaningful attachments to their clinical care team. PKLDs' assumption then can be that all dialysis units and staffing are the same. ∐ PLKD on home therapies previous routines/friendships can change after they start home dialysis. PLKD can become isolated, and can lead to developing anxiety and depression. ∐ Some patients choose UHD as they live alone or in unsuitable housing. ∐ Universal credit and PIP is not currently set up to support people with ESRD. For example, people on unit dialysis sometimes decline a transplant as they are worried what it will do to their benefit claims; also, people who need to make a decision about future treatment are so concerned about setting up their benefits/paying bills that they put their healthcare needs last. Water rates and services often do not account for people on HHD—which needs a lot of water.	∐ PLKD utilize the service as intended, not to fulfil unmet social care needs. New services are developed/modified to address unmet social care needs (e.g. isolation, loneliness, home care and social support). ∐ PLKD should be supported onto a home therapy pathway early and not base decisions on the familiarity of clinics/and clinic staff. ∐ Each local authority is accessed for its capacity to manage PLKD including numbers, resources and link staffing. ∐ Additional social services are more routinely accessed e.g. community connectors, red cross, mens sheds, lunch clubs, Dewis Cymru – which can identify different groups of people to support social care and social prescribing services. ∐ GP services and sign posting are more routinely used. ∐ Tailored home care packages for PLKD are offered. ∐ PLKD are offered a holistic assessment when they come into the service and this includes social care needs. ∐ New roles are developed in the community such as ‘well‐being practitioners' or ‘support practitioners' for the purpose of supporting Home therapies of patients. ∐ Resources and time are spent upskilling nurses to sign post to these services. ∐ The home therapies teams host regular newsletters and updates to sign post to community activities that do not necessarily need to be based on dialysis or kidney disease. ∐ Barriers to home therapies are identified early, and patients and full MDT teams work together to address practical and psycho/social barriers to home therapies (e.g. unsuitable housing). Options for care and support at home are explored with social service sector and link workers. Option to move home is presented as last resort and if presented PLKD can stay in local areas. ∐ People with ESRD are provided with appropriate entitlements to support them over their lifetime irrespective of what treatment they are on.	∐ Expert PLKD advisors and peer‐to‐peer support workers can provide input to help design more integrated health/social care services that work for people with kidney disease in Wales over their lifetime. ∐ PLKD on home therapies can present to the social services sector with support from renal social workers about the need for linked workers/community connectors in local authorities across Wales. ∐ PLKD stories and experiences can support and develop shared understandings of fears and concerns of home therapies and how this might be different across Wales in particular for those not automatically deemed ‘ideal for home therapies' e.g. living alone, older. ∐ People with kidney disease can support the creation of business cases to work more directly with social services to develop a system that works better for patients with ESRD over their lifetime.
Expectations versus reality	∐ Treatment outcomes do not always match the goals and expectations of patients. ∐ Once patients start on UHD, they are unlikely to move to a different treatment option and some patients sometimes regret their decision to start UHD.	∐ Patients and multidisciplinary teams work together to develop shared understandings of goals, preferences and expectations. Opportunities to revisit this are presented throughout PLKD treatment plan. ∐ Patients on UHD who are suitable for home therapies are given opportunities to review their decision. Treatment switches can happen in a timely way with minimum burden.	∐ PLKD and support groups can work with health and social care professionals to produce advice and guidance about what to expect and how to overcome barriers with treatment burden. ∐ Peer‐to‐peer support and visits to units from PLKD networks can help to unpick the barriers to home therapies and provide key support to switch.
Family members, close friends, unpaid carers			
Influences and concerns	∐ The family often (as with PLKD) often do not want to think about disease progression and future treatments. Sometimes, they struggle to come to terms with disease progression. ∐ The family often has more of an influence on the decision making than the PLKD. Adaptations to the home, anxiety about ‘treatment going wrong’ are common concerns and enough to stop PLKD choosing a home therapy. ∐ The family (especially in the early stages) worry more than the PLKD, but they do not have the same support as the PLKD. ∐ The shifting/changing roles of the family into ‘carers’ are not routinely supported or recognized. The family members often don't get the care and support they need and have little respite or opportunities to discuss their unmet needs.	∐ Family visits are encouraged to discuss their needs and concerns looking ahead. ∐ Novel approaches to home adaptions are shared early; families have the opportunity to discuss concerns about home adaptions and any issues with their perceptions of safety. ∐ Care and support is offered equally to the family as they progress with PLKD into RRT.	∐ Peer‐to‐peer support and groups can be set up with family members, close friends to support the family and focus on the family members' needs and concerns.
Professionals: Multidisciplinary teams			
Bias	∐ Bias in multidisciplinary teams. Some professionals may prefer certain treatments over others, for example, peritoneal dialysis (PD) is currently not discussed as an option with 30%–40% of patients in Wales, but 10%–15% of all patients in Wales are eligible for PD. ∐ There is a lack of acceptance of standard of evidence of benefits of different types of dialysis. ∐ It is sometimes a case of ‘better the devil you know’; some clinicians just default to the history of ‘their unit’ and cannot easily see a pathway outside of that. ∐ There are Inconsistencies/disagreements on who is eligible for home therapy, for example, comorbidities, frailty and low quality‐of‐life (QoL) score. ∐ Regional variation across Wales in terms of what treatments are available, offered, discussed and subsequently chosen across Wales, for example, 50% of frail patients over 70 chose maximum conservative management in one region, but in others, the figure is as low as 9%.	∐ Full multidisciplinary team meetings are held regularly to minimize conscious and unconscious potential bias. ∐ Audit data are presented to MDTs regularly. ∐ Updated research is shared and assimilated e.g. ‘Prepare for kidney care' and other relevant data sets. ∐ Welsh renal professionals receive regular and up‐to‐date training regarding frailty scores and QoL training. MDTs could review patient data and agree on reporting and consistency. Audit data are monitored for consistency. ∐ Reduce or eliminate variation in availability of treatment options across Wales. ∐ MDTs involved in educating PLKD about options receive up‐to‐date training on different treatment options.	∐ PLKD and support groups can help make MDTs more aware of the impacts of conscious and unconscious bias and work together to address it. ∐ PLKD can recollect their experiences of learning about treatment options. This can support MDTs to continue to present and discuss options in a suitable format for each patient. ∐ PLKD can use their influence to ensure sharing and feedback of information on patients between ISPs and NHS. ∐ PLKD and wider key stakeholders can support a redesign of patient education materials, utilizing their regional knowledge and recent experiences. ∐ (note that patient‐specific details cannot be shared outside of the NHS and lack of resources and staffing is not easily changed through CoPro).
Variation in practices	∐ There are regional and local variations in paper‐based educational materials. ∐ There are regional and local variations in the ways in which education is delivered, for example, nurse‐led patient groups, peer‐to‐peer support networks, patient‐led education sessions. Multidisciplinary team (MDTs) meetings, shared decision making tools.	∐ Education materials are pan‐Wales, with consistent messages. Individual units and professionals can tailor core materials as they see fit into their patient education programme. ∐ Clinical nurse specialists across Wales work together to pool knowledge and resources to deliver a varied MDT and patient‐centred education programme. ∐ Multiple learning strategies (video, online, social, peer‐to‐peer, paper based) are deployed to support patients. ∐ Education is focussed on patient experiences first and not on ‘the business' of dialysis.	∐ PLKD and wider key stakeholders can work with Clinical Nurse Specialists to support professional practice and vice versa to improve the consistency of education whilst at the same time providing best care for patients. ∐ Charities can work together to help NHS develop education materials, reduce duplicity and ensure that patients are not overloaded with information and instead education is tailored to patient preferences from the outset. ∐ Patient stories, blogs and resources are used as the templates in which education is built around. Expert patients and family members can be supported/employed to coproduce education materials.
Staffing and training	∐ Not all predialysis nurse specialists are fully trained in the available home therapies. ∐ Some AHP posts in remain unfulfilled for long periods of time, for example, clinical psychology and renal social workers. ∐ Not all professionals feel comfortable discussing disease progression, prognosis or are trained in ‘difficult conversations’, for example, ACP.	∐ All predialysis clinical nurse specialists are up‐to‐date trained (with review) on various home therapies. ∐ Renal posts are advertised and filled in a timely way. Benefits of job positions are advertised widely, highlighting potential career pathways and diversity of working in renal health and social care. ∐ Up‐to‐date training is provided for all Multi‐Disciplinary Teams. ACP ‘experts' are identified across regions.	∐ PLKD on various types of home therapies can be invited to training to share their most recent experiences and learning and support in keeping training and reviews up to date and current with patient experience. ∐ PLKD can share their views on the pros and cons of ACP conversations with the MDTs.
Organizations: NHS, Wales Renal Clinical Network (WRCN), Kidney Charities, Independent Service Providers (ISPs), Welsh Government			
Configuration of services	∐ PLKD's first experience of dialysis tends to be in a unit or around unit dialysis. ∐ PLKD coming into secondary care often have little or no understanding of their disease condition. ∐ Currently, most people only meet their ‘home therapies' teams when they are ready to start on a home therapy. ∐ Palliative care services are not currently part of the renal service and renal nurses are not trained in end‐of‐life care. People who chose conservative management are not getting access to specialist care palliative care services. ∐ There is a lack of dedicated ‘training areas’ for people who are on a home pathway or additional training areas for people who are uncertain/worried about home therapies, for example, option to try nocturnal dialysis, or self‐needling, and so forth. ∐ There are three independent service providers (ISPs) in Wales. Each provider has different focusses in terms of dialysis modalities. ∐ Most units are run by ISPs who specialize in different treatments and they do not all currently offer all options for home therapies. ∐ Many MDTs are unaware of the services that kidney charities provide and do not routinely sign post to them.	∐ Opportunities are taken to discuss dialysis options and choices at home, outside of the unit in an informal setting (E.g. café, meeting room and at home). ∐ Transitions from primary care to secondary care are more clearly defined from the patient and professional perspectives. ∐ Home therapies teams are introduced early as part of the patient overall care team ∐ Primary care/geriatrics/palliative care services are more integrated into renal secondary care services. ∐ Every training centre in Wales has dedicated ‘share care' and ‘share care to home' areas in centre. ∐ ISPs work with NHS, WRCN and the Welsh Government to support targets of 30% on home therapies. ∐ Ensure the consultant role on the unit is visible and is able to champion for home therapies. ∐ Kidney Charities work to ‘join up' different parts of the service (ISPs, Policy contexts, MDTs, NHS) to ensure that the home‐first agenda is followed and supported. Opportunities to raise awareness of services that can support patients chose home, or live well at home are provided.	∐ PLKD can work with professionals to create photo books, videos, virtual realities of home therapies as well as share their stories of home therapies with people predialysis. Charity providers can work to create home training areas that look and feel more like a home and a home environment. ∐ Renal Charity providers are also signposted as part of the patient home care team ∐ Charity providers and PLKD representatives can use their experience, expertise and creativity to design and develop appropriate dedicated training areas for pathways to home. ∐ ISPs share their barriers and enablers to home therapies with the WRCN. Outcomes are fed into the overall service design. ∐ Kidney charities work with the various organizations (including wider sectors below) to (a) identify areas of need, (b) barriers to home therapies and (c) agree on and co‐ordinate plans to implement them.
Limited resources	∐ For the first time in 10 years, the WRCN was forced to request a net increase in investment from NHS Wales to sustain the dialysis service. ∐ Currently, the demand for UHD is outstripping supply.	∐ The WRCN can invest funds to meet the needs of the whole service including social care and well‐being. ∐ It is about asking who ‘should be treated in hospital' as much as asking ‘who is suitable for home therapies'.	∐ PLKD can map out what, where and how the WRCN could invest in the CKD service as a whole, using their experiences mapped against the service delivery. ∐ PLKD and professionals can work together to promote the benefits of home therapies over UHD. Peer‐to‐peer support workers can help identify any unmet social care needs of patients on UHD and work with renal professionals and the social care sector to address them.
Policy contexts	∐ Increasing home therapies is a Welsh Government prudent healthcare policy, but the numbers have remained static. ∐ WRCN target for patients on home therapies is 30%. It was 20% from December 18 to May 19 (6 months). This is the highest it has been.	∐ Welsh renal professionals and PLKD understand the importance and value of prudent healthcare on the complete CKD service. ∐ All renal professionals are aware of WRCN targets; they are monitored and ongoing opportunities for shared learning are made available.	∐ Patient and carer advocates can work to promote the importance of prudent healthcare and what it means for them, that is, benefits of shifting costs or reducing spending in one area. ∐ WRCN targets are higher than NICE guidance. Patients and professionals can promote the Welsh service as an exemplar of home therapies nationally.
Clinical recommendations	∐ NICE guidance now (2018) states that conversations about renal replacement therapy should start one year before needing to start a treatment, but it is not easy to monitor CKD progression.	∐ The impact of earlier conversations is monitored and compared with uptake of home therapies.	∐ PLKD and family members who started RRT a year in advance can share their stories at WRCN meetings for shared learning.
Outside secondary care including early‐stage CKD, the public, wider health and social care services			
Overall Health literacy	∐ Many people in Wales do not know they have Chronic Kidney Disease until they go into renal failure.	∐ GPs, other health providers and the social care sector are more aware of general kidney health.	∐ Kidney charities, related third sector organizations and wider stakeholders can work to promote kidney health across Wales.
Population	∐ Wales has a sicker and older population than England.	∐ CKD renal services in Wales are designed to meet the needs of an older and sicker population than England. Wales should also be a healthy place; we should work with wider public health services to make Wales a more healthy population.	∐ Encourage general awareness and promotion of kidney health and earlier interventions such as social prescribing. (note that we cannot change the current demographics through coproduction).
Wider service configurations	∐ There are limited resources in the wider health and social care services. ∐ There are insufficient deceased donor kidneys available for transplant.	∐ Resources are reconfigured/re invested into key identified social care services to support PLKD's and family members' unmet social care needs and ensure that these needs are not barriers to choosing a home therapy. ∐ Welsh CKD services should continue to promote and take opportunities to promote organ donation registration.	∐ Involvement of social services and PLKD can help identify more quickly the social services which could potentially be invested to support uptake of home therapies and best patient care. ∐ Transplant recipients and people waiting can work with WG to share their story and register as a contact with comms teams.
Research	∐ There is slow progression with new treatments.	∐ Opportunities for new research including clinical trials and opportunities to work together to reduce time on treatment development and maximise progress are taken. New treatments should result in fewer people needing dialysis, early detection and prevention of disease progression. Research funding is directed to kidney disease.	∐ PLKD involvement in research should be embedded into routine care. PLKD can share their research experiences and encourage more people to get involved.
Culture	∐ Culturally, we are not good at Advance Care Planning; we do not like to talk about death and dying.	∐ We have conversations about end of life as part of routine healthcare. Campaigns such as Organ Donation awareness and Dying Matters are tapped into to encourage people to talk more openly about their end‐of‐life care pathway.	∐ Related charity providers such as CRUSE, Dying Matters and Donor Family Network could be partnered with to support campaigns to talk about end‐of‐life care.
Public perceptions and attitudes	∐ People living with kidney disease face huge stigmas including attitudes of laziness, being accused of drug addiction and general lack of understanding of disease burden.	∐ General awareness and knowledge of kidney disease risks, burdens and treatments are improved across Wales.	– Kidney charities can work with wider charity providers and Public HealthWales to support raising awareness of ESRD treatments.

Abbreviations: ACP, advance care plan; CKD, chronic kidney disease; ESRD, end stage renal disease; GP, general practitioner; HHD, home haemodialysis; ISP, independent service provider; MDT, multidisciplinary team; NHS, National Health Service; NICE, National Institute for Health and Care Excellence; PIP, personal independent payment; PLKD, people living with kidney disease; QOL, quality of life; RRT, renal replacement therapy; SDM, shared decision making; UHD, hospital‐based dialysis; WG, Welsh Government; WRCN, Wales Renal Clinical Network

The research team reviewed Table 2 together at their weekly core team meetings and discussed and further refined the evolving content. The team hosted a specifically co‐ordinated data analysis meeting (13 November 2019) with selected wider stakeholders including people with kidney disease who were becoming increasingly knowledgeable on the sustainability issues. Table 2 was cleaned, and each sustainability element was summarized and sense‐checked by the multidisciplinary research team and people living with kidney disease and presented as a final stand‐alone sustainability table (Table 2).

**Figure 3:**
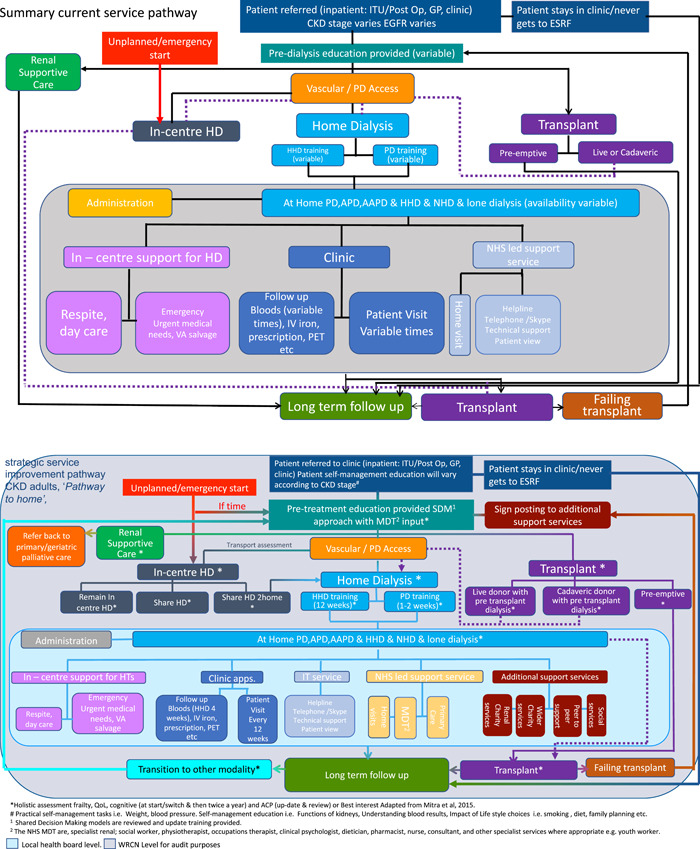
Draft service improvement documents ‘Pathways to Home’

2. To redesign service pathways in adult CKD

- Data collection
  The original draft of the service improvement pathway 'pathways to home' (Figure 3) was designed by the lead kidney nurse for Wales, also a study coapplicant and expert in the current state of Welsh kidney services. The initial drafting also had patient input from representatives of the WKRU.
  The research team worked to ensure that the draft pathways were shared with representatives from the full NHS MDTs; the specialist renal nurses in predialysis and home therapies, professionals allied to medicine including physiotherapists, dieticians and clinical psychologists, kidney social workers and youth workers, people living with kidney disease, family members, kidney third sector CEOs and staff working for kidney charities, and volunteer peer‐to‐peer support workers—many of whom were either living with kidney disease or a family member of someone with kidney disease. This took place over 16 months from March 2019 to June 2020.
  We did this by sharing the first drafts of the pathways at quarterly NHS specialist nurse meetings and quarterly professionals allied to medicine meetings (which were also attended by patients and kidney third sector partners). The research team also worked with kidney charity partners to set up specially coordinated Zoom meetings to present to people living with kidney disease, family members and charity provider executives. These Zoom meetings were developed into webinars, which were made available online on YouTube and promoted on social media (Twitter, Facebook, mailing lists) for people to refer and feedback into via comments, telephone and email communications. This process continued over 15 months at face‐to‐face and virtual meetings until no new options or additions were forthcoming and there was a sense of data saturation.
- Data analysis

Analysis was integrated into the development of the pathways from the outset. We presented the current service pathway as a backdrop and then asked what needed to/should be added to improve uptake of home therapies. Each engagement event was an opportunity for refinement and clarification from the multiple perspectives. This helped to highlight gaps in terms of what the service did not currently have and then move onto possibilities for improvement across the multiple service pathways. The before and after snap shots helped set out the vision for what kidney services could look like at a fairly high level across Wales and to more easily present this in relevant ways to the multiple stakeholders for their input.

**Summary of key events and attendees**

**All Wales National Home Therapies meeting** (29.04.19)**:** Total *n* = 20, *n* = 18 renal professionals (kidney specialist nurses, nurse managers and directorate managers), *n* = 1 family member and kidney charity representative, *n* = 1 people with kidney disease

**Presentation and meeting with Kidney patient ‘befrienders’ via kidney charity** (12.09.19)**:** Total *n* = 10, *n* = 2 family members, *n* = 8 people with kidney disease

**All Wales Health and Wellbeing reference group** (22.10.19)**:** total *n* = 10, *n* = 9 renal professionals (dieticians, social workers, physiotherapies, psychologists) *n* = 1 kidney charity provider.

3. To review patient education materials and processes

- Data collection
  There were around 15 specialist nurses working across five kidney centres in Wales. We contacted each centre lead nurse and requested that they collate their current paper‐based education materials and send to the research team. We developed a simple pro forma education flow chart and requested that the nurses fill it in to illustrate their current education practice, from initial conversation through to making a choice of RRT. We also requested that all Wales figures for numbers of people on home dialysis from the WRCN were shared. The research team then invited the lead nurse of the only centre that was achieving the national guidelines of 30% of patients on home dialysis to come and present at a specially co‐ordinated coproductive multidisciplinary meeting (with people with kidney disease and family members in their service also attending) and explain how their service worked. Academic members of the research team were also invited (following a study update at an NHS quarterly meeting) to attend a house visit with a specialist nurse (from a different centre) to observe their initial conversations with a person needing renal replacement therapy and their family members.
- Data analysis

After the materials were collected, the research team reviewed their content alongside a review of the numbers of people on home dialysis across Wales by region and centre and presented the review as well as examples of education materials back to a group of professionals, people with kidney disease and family members for their input. Despite education processes looking broadly similar across centres, on paper, the review of the number of people on home dialysis varied significantly (File S3). The lead nurse presentation was followed by a question‐and‐answer session to an MDT room of practitioners from different services, people with kidney disease and family members to ask more about why their service appeared to be overachieving. People with kidney disease and family members were also invited to share their recent experiences of predialysis education and expressed their views on the barriers to home therapies from their perspectives. The field visit to the pre‐dialysis person's home with their family was followed by a detailed discussion with the nurse, a report and presentation of findings to the research team at core team meetings.

**Summary of key events and list of attendees**

**National Chronic Kidney Disease specialist nurse meeting** (22.11.19): Total *n* = 13, *n* = 9 kidney professionals (specialist kidney nurses), *n* = 2 family member, *n* = 2 people with kidney disease

**Presentation from home therapies teams** (25.11.19): Total *n* = 35, *n* = 12 kidney professionals (specialist nurses, nephrologists, social workers), *n* = 10 family members, *n* = 13 people with kidney disease

**Field visit with CKD nurse to home consultation** (27.11.19) total *n* = 3, *n* = 1 specialist kidney nurse, *n* = 1 family member, *n* = 1 person living with kidney disease

**Visits to dialysis Units across Wales** (25.02.19, 29.08.19, 30.08.19) total *n* = 17 people with kidney disease

4. To better understand patient and public attitudes towards the costs of kidney care and services

- Data collection
  Academic members of the research team attended nonkidney disease‐related public events during the 18‐month data collection window of the study. This included hosting a market stall at the St. David's Day parade, presenting at the all Wales Involving people festival and attending local community group meetings with Black Asian Minority Ethnic individuals and groups to enhance opportunities for learning and interactive feedback with the public in Wales including minority and underrepresented groups. We attended the National Transplant games in Newport and the WRCNs ‘Renal Roadshow’ with a banner and preamble to introduce the topic of treatment option costs to the public and invite their opinion. We delivered presentations at kidney patient‐led conferences and meetings to discuss costs and gather perspectives with larger and smaller groups of kidney patients. We also approached social services commissioners in Wales to learn from their perspectives and invited commissioners to a specially co‐ordinated meeting on Zoom to discuss the sustainability of kidney services.
- Data analysis

Analysis was built into the discussions with members of the public. We asked about their perceptions of costs and asked them to list which treatments they felt were the most to the least expensive. This was followed by opinions on service configurations and, in particular, if and how they felt social services could support a home‐first agenda. Field notes were collected from each engagement and presented back to the research team at core meetings for discussion.

**Summary of key events and list of attendees**

**St David's Day parade Pembrokeshire** (01.03.19): Total *n* = 40 members of the public

**Bangor University Centre for Health Economics invited seminar** (14.05.20): Total *n* = 15 members of the public

**‘Renal Roadshowz’ West Wales** (04.07.19): Total *n* = 40, *n* = 20 kidney professionals (specialist nurses, physiotherapists, dieticians, pharmacists), *n* = 10 family members, *n *= 10 kidney patients

**Workshop with kidney ‘befrienders'** (12.11.19): Total *n* = 9, *n* = 1 family members, *n* = 8 people living with kidney disease

**‘Home therapies café’** (07.02.20) total 9, *n* = 6 people with kidney disease, *n* = 3 family members.

## 3 FINDINGS

1. Creating a vision of a more sustainable adult kidney service in Wales

People living with kidney disease could play a potentially vital role in addressing specific sustainability issues (such as professional confidence in having perceived difficult conversations e.g., Advance Care Planning) all the way through to policy contexts and updating clinical practice (Table 2, What can be changed through coproduction). Involving people living with kidney disease and available peer‐to‐peer support networks earlier to help explain the benefits of home dialysis, alleviate concerns and empower people to make informed decisions about future tailored treatment plans could encourage more people to opt for home dialysis (Table 2, Decision making).

There needed to be more options to ‘try home dialysis out’ such as dedicated home training areas without any perceived burdens such as wait times including ‘nocturnal hotels’. The visibility of home‐based dialysis needed to increase across Wales and throughout the service (Table 2, Configuration of services).

The clear and known barriers to people opting for home dialysis, for example, needling, home reconfigurations, living alone, etc could be eliminated with creative and personalized approaches from the MDTs. We frequently found that factors listed as barriers were not particularly difficult or problematic to address or overcome such as worries about self‐administration of treatment, perceived inconveniences of home reconfigurations or perceived time saving of hospital dialysis (Table 2, Influences and concerns). Social care services needed to be better integrated with kidney health services to directly address where the NHS was picking up areas of unmet need, which might be better placed elsewhere. Healthcare services needed to recognize that CKD is a lifelong condition and that the service needs to be able to adapt to people as they progress through their life as well as their disease condition (Table 2, Social contexts and services).

2. Redesigning service pathways in adult CKD, pathways to home
  Required modifications to existing care pathways clearly showed that there needed to be more defined pathways for people ‘to get home’ and more quickly (Figure 3). The pathways highlighted areas where the service was potentially picking up areas of unmet need that might be best placed elsewhere; for example, patients on a conservative management pathway were currently being managed by specialist *dialysis* nurses and potentially missing out on wider palliative care services.
  We observed a lack of support services for both patients and family members in these cases (clinical, psycho/social, wider social care support) and uncertainty within the renal teams with how best to manage them as a result.
  New stepped stages in the pathway introduced opportunities for more MDT input, patient‐led support interventions and signposting to wider third sector and social services across all pathways. This could help reduce or prevent patients being siloed or treated solely on the basis of their current kidney treatment. It would also seek to eliminate service configurations that saw people finding themselves by default on unit/hospital dialysis with no clear alternate pathways (Figure 3, New pathways).
  Pathways not clearly associated with home dialysis, for example, transplant were having an influence on people opting for or currently on UHD. For example, most people awaiting transplant in Wales are on UHD, but we know that most transplants will eventually fail and that patients rarely decide to switch dialysis modalities (Table 2, Decision making). These new pathways included more clear definitions and acknowledgement of the multiple ‘pre‐transplant dialysis’ pathways and their potential influence on getting onto home dialysis.
3. Review of patient education materials and processes
  We found that the attitudes of professionals had a huge influence on uptake of home dialysis, and there was variation in perceived barriers to home dialysis and how people felt they could overcome them. The clinical nurse specialists enthusiasm for home dialysis and a very connected service both in practice (they work in the same offices) and in behaviour (all of the team felt no barrier was too high to overcome to get people on home dialysis) helped to create a culture of a home‐first service. Many of these nurses even felt that the national targets were too low. They also expressed concern that It was too easy for people to opt for UHD nowadays, and that the pathway had become the norm rather than the exception in Wales. This particular team had developed over time a home dialysis‐led service with problem‐solving approaches to known barriers to home dialysis (e.g., needling, living alone, space, home reconfigurations, safety etc.), of which they were very proud. Close working relationships and up‐to‐date expertise in the various dialysis treatments helped create a culture that questioned the status quo and a desire to change it (presentation from home therapies teams (25.11.19)).
  Paper‐based education (based largely on describing dialysis and kidney disease) was having little impact. Patients rarely read the resources provided. Family members did, but this only exasperated patient and family member concerns and increased worry. There was also little evidence of resources especially developed for family members. In service configurations with high numbers of patients on home dialysis, the family were given equal attention by professionals and managed alongside, but separate to patients (field visit with CKD nurse to home consultation (27.11.19)).
  Professionals rarely, if ever, used predesigned paper‐based education materials as part of their conversations. They preferred to rely on their own expertise and rapport with the patient. During the study, some professionals began to reconsider what ‘education materials’ actually mean and how they can support a home therapies agenda. This began by shifting the purpose of education away from explaining dialysis and more towards showcasing living well on dialysis. People going through kidney replacement therapy education will always have a consultant and specialist nurse to manage their care and will have access to a wider MDT for more specific needs. Education programmes are needed to help and support these roles, signpost to their expertise and then fill gaps in professional knowledge, for example, lived experiences as told by patients. Peer‐led networks and opportunities to interact with more patients on home dialysis were considered essential, as was removing and decommissioning any information and literature that did not talk to a home‐first agenda (Table 2, Variation in practice).
  While most professionals said that they supported home therapies, there was variation in perceived barriers to home therapies and how people felt they could overcome them. This was exacerbated by a lack of clear processes for ‘escalation’ for complex cases such as sudden and unexpected change of mind, decisions that were firmly against clinical (and family) advice or cases where there was disagreement (amongst professionals) as to what treatment to recommend (Table 2, Bias).
  Finally, we also observed that a standard model of dialysis was deskilling some of the specialist workforce, for example, up‐to‐date knowledge of various types of dialysis, confidence in (re)approaching families who initially declined home dialysis or changed their minds and overall experience in communication techniques including SDM (Table 2, Staffing and training).
4. Patient and public attitudes to costs of health services

There were substantial gaps in peoples' understandings of the costs of dialysis treatments as well as mixed views about shifting or reconfiguring costs. While in general patients and members of the public understood that NHS services cost money, they had not previously thought about the different costs of various treatments. People were very surprised to learn about potential variations in costs and the potential savings. Very few people had previously considered the fact that the NHS might be picking up unmet social care needs, which were in turn having a substantial impact on NHS finances. Once members of the public and patients were made aware that the potential savings were substantial; they felt that everything possible should be done to realize cost savings and develop more sustainable services (Table 2, Policy contexts).

We also observed clearly that policy contexts designed to develop and create more fair, equitable and parity to public health service spending were not well understood. Sometimes, discussions about costs and service reconfiguration were met with suspicion and an overall perception that potential saving was equated to cost‐cutting. Responses to shifting costs between services were mixed. The public generally supported the idea of shifting costs from social services to health, but not the other way around. The public perception was that social services were stretched to an upper limit and would be unable to cope with any costs newly attributed to social services. Social service workers, volunteers and commissioners initially struggled to see some of the unmet social care needs that the NHS was picking up and the potential costs of this. Their immediate reactions were that dialysis is a primary healthcare need, and they did not necessarily see the potential benefits of a more integrated health and social service package of care to pick up very clear unmet social care needs, for example, isolation and loneliness (Table 2, Outside secondary care).

Costs were never a consideration when discussing and recommending treatment options to patients. Professionals do not routinely discuss costs and have limited knowledge of NHS costs, spending, commissioning and service design. Importantly, we saw that professionals did not feel at all comfortable talking about costs and service configurations in front of patients and actively avoided talking about real barriers in terms of service delivery in general with patients (Table 2, Limited resources).

## 4 DISCUSSION

### 4.1 Main findings

This study highlighted the *effects* of the unsustainable service on the multiple stakeholders. This included an unhelpful standardized model of care for all people with kidney disease, a default position to UHD as the norm and a deskilling of some specialist key roles. Key stakeholders—people on home therapies—are essential to understanding sustainability contexts and developing new service pathways, and their input remains essential to realizing the outcomes and ambitions of any coproduced outcomes. More integrated health and social care kidney services have the potential to lead to a more financially sustainable NHS (reducing NHS burden by reconfiguring services out of hospitals and more towards home), but this needs to be underpinned with a shared vision from professionals in key influencing roles if models of care are to switch from acute to long‐term sustainable care packages. A significant gap was the lack of understanding (from the multiple perspectives) of dialysis monetary value—how much various services cost and how they are delivered. Policy contexts (e.g., value‐based and prudent healthcare) were not well understood; generally, people understood potential service improvement initiatives as a cost‐cutting measure and could see (at least initially) little potential benefits to them. Many healthcare professionals did not immediately connect potential impacts of UHD on long‐term patient outcomes, especially nonclinical impacts, for example, restrictions on travel, work, childcare and lifestyle. Many had never heard of the policy contexts or were unclear as to how these related to them.

### 4.2 Meaning of this study in relation to other research

As with previous research, we found that local practices or perhaps more accurately ‘individual personalities’ influence the uptake of home dialysis and help explain some of the observed variation between centres.^52^, ^53^, ^54^ This study went a step further in terms of realizing how these individuals can be (re)imagined as a resource—key influencers to change—at the multiple levels from practice through to policy.

Generally, previous research into kidney health service improvement has a high focus on clinical outcomes.^55^ More recent health service improvement initiatives have focussed on encouraging people already on UHD to have a more proactive role in their dialysis and increase opportunities for self‐care.^56^ In this study, the coproductive approach set within the context of sustainability—and not just the clinical benefits of home dialysis—supported key stakeholders to unpick on their own terms what they saw as barriers to home therapies and ways to achieve more sustainable services.

The coproductive *outcomes* in this study reinforce the assertions of Elwyn et al.^23^ that SDM on its own does nothing.Interventions intended to bring about change need appropriate infrastructures, training and multiple linked networks to realize their aims. The coproductive *approaches* in this study have helped to identify what resources are already available and where additional investments may be needed. This contributes to the growing literature around global coproduction practice and value.

### 4.3 Implications for clinicians and policymakers

Increasing the number of people on home dialysis is a global health priority. The UK NICE first recognized the benefits of home dialysis more than 20 years ago;^57^ yet, unit/hospital dialysis continues to increase. Lord Nigel Crisp (independent Member of the House of Lords), in a recent letter to the BMJ, highlighted that ‘UK health and care system, like all others in the West, is still largely using a 20th century acute care model of service delivery to meet 21st century needs’ and put forward seven factors necessary to contribute to sustainability (*1. Efficiency and effectiveness of health and care provision, 2. Availability of well‐trained health and care workers, 3. Costs and economic benefits, 4. Health and resilience of the population, 5. Contribution of carers and informal networks of care, 6. Integration of policy and practice with other sectors and building healthy and health creating communities *and *7. Public and political acceptability and support),* highlighting that two in particular need more emphasis—economic benefits and multisectoral partnerships. This study unpacked key issues within all seven factors (Table 2), and by utilizing a coproductive approach, highlighted the ways in which the current service was working contradictorily to prudent healthcare and created a new vision for what a good adult CKD service looks like in Wales.^58^

Commitment is needed from all the multiple stakeholders to develop networks and opportunities for meaningful knowledge coproduction and ways to sustain it. This includes working more closely and more frequently with people living with kidney disease, their family and networks of support. As of April 2020, there were 292 people on various types of home dialysis in Wales. This group are potentially one of the biggest untapped resources that can influence changes in attitudes and culture towards a home‐led service and actually support (rather than compete with) the wider health and social care agenda towards prudent healthcare.

Knowledge gained from this study includes the coproduced vision of more sustainable services, improved pathways to home dialysis and opportunities for greater integration of social care services and highlights ways to more proactively involve people with kidney disease more directly in service reconfiguration. This learning and the methods to coproduce it have the potential for transferability to similarly configured global healthcare systems. Redesigned pathways may even be more easily adapted outside of Wales, which has an older, sicker and more deprived population than other countries, and health literacy is generally low. Although additional account may need to be taken of country‐specific social care systems and the ways in which they currently integrate with health services to make best and better use of existing resources.

### 4.4 Strengths and weaknesses of the study

This is the first all‐Wales co‐productive study to address the sustainability of adult CKD health and social care services from direct and indirect key stakeholder perspectives. We believe that this is the first application of the principles for knowledge coproduction in sustainability research in kidney health research and can be built upon in future research, quality improvement and service development initiatives.^50^ The study was limited to Wales, which has a predominantly white population, and it was not able to account for extraordinary events, for example, COVID‐19 (data collection completed just before the pandemic), although many feel that COVID‐19 has simply provided a stark reminder of the unsustainability of the NHS in general in its current guise and the need to keep people living well and at home. Finally, this study was not designed to measure any specific outcomes of sustainability, but rather the potential of coproduction to improve sustainability for the multiple stakeholders.

### 4.5 Unanswered questions and future research

The question can coproduction lead to more sustainable adult CKD services in Wales was the focus of this study. In this context, we have only been able to partially answer it. We do not know explicitly whether coproduction will lead to greater sustainability of kidney services as this was not conceptualized as a longitudinal study to monitor behaviour change over time. For this, more research is needed over a longer period, with a larger and more diverse population to build upon the work outlined in this study, which includes templates on ways to work coproductively with NHS MDTs, kidney charities and people living with kidney disease. The NHS now needs to implement the new clinical pathways and embrace the transformational roadmap to change that was co‐produced with patients.^44^ Going forward, it will be important to evaluate the outcomes of coproductive research and processes using routine data collection methods, for example, routinely collected patient data, health economics modelling, patient‐reported outcome measures and patient‐reported experience measures.

## 5 CONCLUSION

Coproductive research helped start a conversation between key stakeholders and researchers about sustainability. Much more needs to be done to increase the overall understanding of NHS financial and service structures to ensure that this is not a barrier in any future coproduction. Coproduction has the capacity to identify and reverse unintended consequences of health service systems that have (almost always) grown based on perceived immediate need, with little evidence basis and over a very long time. More case studies are needed to provide exemplars of what key policy contexts look like in health service delivery to realize the ambitions of prudent healthcare at scale.

## CONFLICT OF INTERESTS

The authors declare that there are no conflict of interests.

## AUTHOR CONTRIBUTIONS

Leah Mc Laughlin led on coproductive activities, conceptualized the coproduction and drafted the manuscript. Gail Williams co led on coproductive activities, drafted key service redesign pathways, produced home therapies reports, undertook data analysis and data integration and reviewed the manuscript. Gareth Roberts developed the overall study design, co led on some coproductive activities and reviewed the manuscript. David Dallimore contributed to the design of coproductive activities, supported overall coproduction, contributed to data analysis and data integrated and reviewed the manuscript. David Fellowes contributed to data analysis and data integration, and reviewed the manuscript. Joanne Popham provided key coproductive support to deliver objectives, key understanding of configuration of peer support workers and reviewed the manuscript. Joanna Charles supported the coproduction of some activities and supported sharing of interim findings. James Chess developed the overall study design, and supported data integration and analysis. Sarah Hirst Williams provided key understanding of NHS home therapy service configurations, coproduced data and reviewed the manuscript. Jonathan Mathews supported the overall coproductive activities, coordinated multiple group meetings, provided data sets and reviewed the manuscript. Judith Stone provided key coproductive support to deliver objectives, key understanding of charity providers configurations and their potential influences and reviewed the manuscript. Teri Howells supported the overall study design and supported coproductive development. Teri sadly passed away before this study was completed. Linzi Isaac supported the coproduction of activities, supported sharing interim findings with UK patients and reviewed the manuscript. Jane Noyes oversaw coproductive activities, conceptualized the coproduction and drafted the manuscript.

## ETHICS STATEMENT

This study was approved by Wales REC 5. REC No. 19/WA0020. IRAS ID: 255387.

## Supporting information

Supporting information.

Supporting information.

Supporting information.

## Data Availability

The data that support the findings of this study are available in the Supporting Information Material of this article.
