# Supplementary material for: Assessing the efficacy of coproduction to better understand the barriers to achieving sustainability in NHS chronic kidney services and create alternate pathways
Source: Health Expect. 2021 Dec 28;25(2):579–606. doi: 10.1111/hex.13391 (PMC8957730; doi:10.1111/hex.13391)
Supplement: Supplementary file 1 — Supporting information. [file HEX-25--s002.docx]

**Supplemental file 1.**

**Hospital and home dialysis – developments and trends**

The concept of dialysis as a means to treat kidney disease has been around for over a century.^14^ Haemodialysis machines then known as ‘artificial kidneys’ were first used in 1940s to treat acute kidney injuries ^15^ and in the 1960s they expanded to include ‘maintenance dialysis’ for people with CKD. However due primarily to lack of space and high costs associated with administering the treatment dialysis was only available at home.^16^ By 1973 around 40% of all dialysis patients in the U.S were treated on home haemodialysis.^17^ A combination of health, social, political, and economic factors over the following decades saw a decline of home based haemodialysis. These included: increase in transplantation as a more effective KRT, developments in PD, high burden of treatment, healthcare coverage began to include all dialysis, increase in (aging) populations, complications resulting from long term dialysis, and pressure on healthcare services to provide dialysis for all people with kidney failure.^18–22^ Combined these factors led to a global increase in hospital based dialysis which almost immediately reached capacity. The answer was a move towards working in partnership with independent sector providers to provide off site “satellite” dialysis units to meet growing demands and address the known challenges.^23^ This turn from home to hospital/unit dialysis was not necessarily intended, in fact early predictors of dialysis growth and costs over 5 years from 1971-1976 were modelled on a 40% increase in transplants, a 50% increase in home dialysis, and hospital/unit dialysis would stay the same.^24^

Amidst the growth of dialysis units In the early 80s the UK faced fierce criticism for being behind Europe and the U.S in terms its dialysis provision.^25^ But the UK quickly caught up, in 1958 there were 3 dialysis units across the UK, today there are over 386 with many more in planning and development.^26^ By the mid noughties unit dialysis had become so embedded as the routine model of care that almost all service improvement and planning focussed on ‘dialysis c*loser* to home’ rather than dialysis *in the* home.^27^

| **Most common options for home dialysis in the UK** |
| --- |
| **Peritoneal Dialysis (PD)– uses the peritoneum, a natural lining of the abdomen, to remove fluid and waste.**   - Automated Peritoneal Dialysis (APD) – a machine exchanges fluids overnight while the person sleeps. - Continuous Ambulatory Peritoneal Dialysis (CAPD) – a manual exchange occurs several (normally 4) times a day.   **Home Haemodialysis (HHD) – uses an artificial membrane and a machine to dialyse the blood and remove fluid and waste**   - Home Haemodialysis – can be done 3/4 times a week for three to four hours or longer each time. Is flexible can be done more or less depending on persons needs. - Nocturnal Home Haemodialysis – longer, slower gentler dialysis done at night while asleep. |

Given the increase in available types of dialysis most recent research has turned to comparing the benefits of the various treatments from the multiple perspectives. Some early results reported problems in terms of randomisation and selection bias ^28,29^ but increasingly the evidence suggests that home dialysis has better outcomes in terms of survival, quality of life and cost effectiveness. ^30–35^

In spite of the increase in options for home dialysis, in the UK today over 80% of dialysis patients are currently on UHD and most people also start dialysis on UHD (69%). Similar patterns of high numbers of people currently on and opting for UHD compared to home dialysis can be seen throughout Europe ^36^ and the U.S.^37^

However UK trends mirror the global picture which shows that hospital/unit dialysis is increasing and there is large variation in the numbers of people on home dialysis between centres. Studies have concluded it is due to variation in local practice rather than health service configuration issues e.g. budgets, population density, patient profiles or uptake of a different KRT.^38–40^

**The picture today.**

Today in the UK there are 29,064 adults currently on dialysis. In spite of the available options for home dialysis (see box most common options for home dialysis in the UK) 83.2% are on hospital/unit dialysis and only 16.8% are on a home dialysis. Of these, 12.2% are on PD and only 4.6% are on home haemodialysis.^27^ Most people nowadays also start dialysis in a unit or hospital when given a choice. In 2017 68.9% of adults in the UK started on hospital/unit, 21.1% on a home therapy (20.3%PD and 0.8%HHD) and 10.2% had a pre-emptive transplant.^28^ National Institute for Clinical Excellence guidelines estimate that a minimum of 30% of the current dialysis population in the UK could be on home dialysis. However UK trends mirror the global picture: hospital/unit dialysis is on the increase ( See figure Growth in UHD numbers in the UK below) and there is wide variation of people on home dialysis between centres. In 2017 only 8/71 (11%) centres achieved 30% of people starting on home dialysis but no single centre had 30% of people currently on home dialysis.^43^


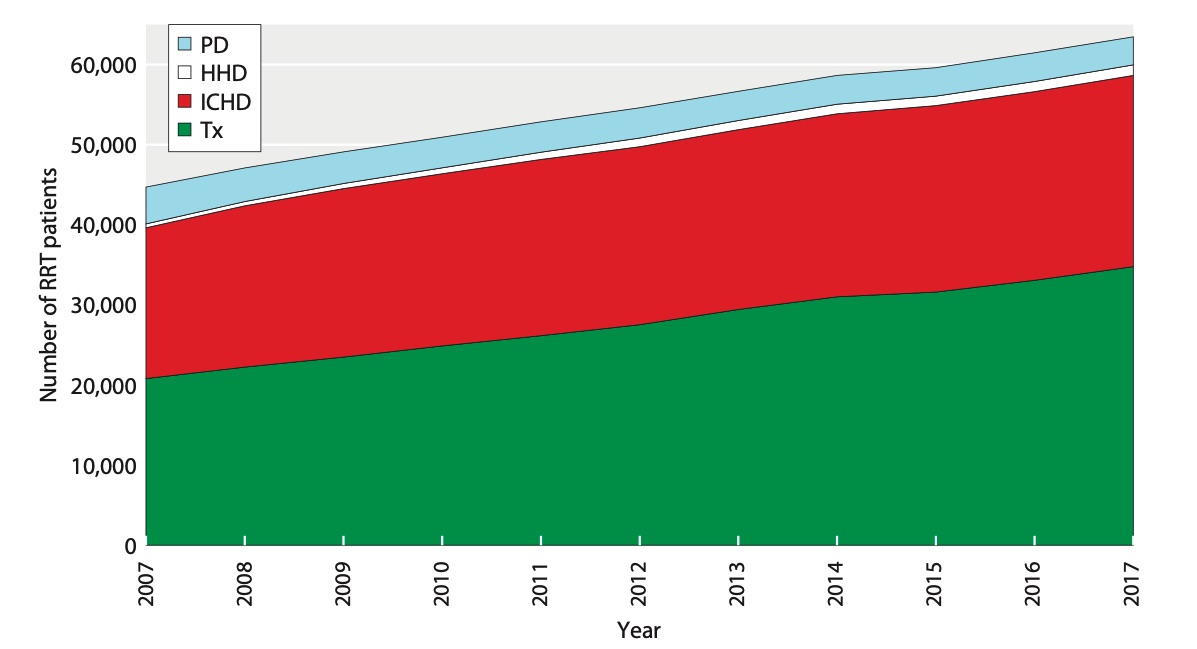


Growth in UHD numbers in the UK 2007-2017.

Key

PD – Peritoneal Dialysis

HHD – Home Hemodialysis

ICHD – In Centre Hemodialysis

Tx – Transplant
