# Supplementary material for: Assessing the efficacy of coproduction to better understand the barriers to achieving sustainability in NHS chronic kidney services and create alternate pathways
Source: Health Expect. 2021 Dec 28;25(2):579–606. doi: 10.1111/hex.13391 (PMC8957730; doi:10.1111/hex.13391)
Supplement: Supplementary file 2 — Supporting information. [file HEX-25--s001.docx]

**Supplemental file 2. UK standards for public involvement, co-productive examples, and reporting guidelines for research**

| **The UK standards for Patient and Public Involvement mapped against the co-production activities** | |
| --- | --- |
| **INCLUSIVE OPPORTUNITIES** | We:   - **Attended BAME community events led by partners Race Equality First** (e.g. intergenerational cooking classes) to include could minority perspectives on study design, recruitment processes and ensure our study materials were clear – even to those whose second language was English; - **Hosted stands at Bingo Halls in Cardiff** to gather input from public groups whose members were more likely to fit our target demographic; - **Recruited all Specialist kidney nurses** to utilise their expertise to recruit a maximum variation of participants from across Wales - **Visited dialysis units across Wales** and spoke to willing people before, during and after their dialysis sessions to input into the research findings and outcomes. - Hosted stalls at renal led events such as the **transplant games in Newport, kidney charity patient conferences in Swansea.** - Hosted stalls and presented at non kidney led events such as the **St. David’s Day Parade in Pembrokeshire, the European Festival of Social Sciences in Bangor, and delivered Ted-style talks at festivals in Wales National Museum.** |
| **WORKING TOGETHER** | We:   - **Set up a multi-disciplinary team** led by kidney consultants, specialist nurses and renal service commissioners whose consultations and experience with patients highlighted concerns about the high numbers of people on hospital dialysis. - **Involved the All Wales multi-disciplinary kidney workforce (**including clinicians, specialist nurses in transplant, home therapies and pre dialysis, psychologists, dieticians, physiotherapists, pharmacists, social workers and youth workers). - **Involved wider health and social care service providers** who could potentially contribute to more sustainable services including patients, family members and kidney charities (Kidney Wales, Paul Popham, Kidney Care UK), wider third sector services such as Carers Wales, Citizens Advice and Action for Elders, Industry partners (who provide dialysis services across Wales – Bbraun, Fresenius and Baxter), local authority and social service representatives to share knowledge and build working networks. - **Hosted All Wales co productive meetings** (five rotated between north and south Wales) to share interim findings, gather feedback and coproduce next steps. - **Coproduced a new vision for sustainable kidney services** in the form of a table with sections to include input from the multiple perspectives. - **Coproduced new service pathways** which included new ‘pathways to home’ and ways the various services could audit and monitor progress. |
| **SUPPORT AND LEARNING** | We:   - Invited patients to become **co-applicants** - Invited patients to **co present at national research meetings**. - Invited key collaborators (nurses, administrators, charity providers, patients, family members) **to co-author academic articles.** - Invited patients, specialist nurses and commissioners to **review and interpret patient interview transcripts** - Provided opportunities for key partners (pre dialysis specialist nurses, and kidney charity providers) to **present at specially curated co-productive meetings** about their services, what works and why - Offered opportunities for NHS co-applicants to **attend patient interviews.** - **Academics were offered opportunity to observe specialist nurses speak to people about their future treatment options.** |
| **GOVERNANCE** | - Patients were involved in: writing the application; ethical approvals; study design; and study management via weekly study meetings, and attendance at steering committees. |
| **COMMUNICATIONS** | We:   - produced a **website, social media (Facebook page), YouTube channel, and monthly newsletter** which included updates, research activities and relevant events. This was emailed to a study mailing list of over 100 and included kidney patients and family members across Wales, hard copies were also sent to the 18 kidney dialysis units in Wales. The newsletter was also distributed through the All Wales Renal Clinical Network an internal renal NHS mailing list of over 200. View these outcomes here <http://www.kidneyresearchunit.wales/dialysis-options--choices> - **Created online video webinars** in response to the COVID 19 crisis to ask for input into draft service redesigns and posted them on YouTube <https://www.youtube.com/watch?v=euIfFW4PjAk&t=518s> - **Hosted online zoom meetings** facilitated by kidney charity partners to continue to gather input from patients during lockdown on research outcomes. - **published in the national patient led magazine – Kidney Matters and in Medi-Wales (The Life Science Network for Wales) ‘Lifestories’ feature.** |
| **IMPACT** | We:   - jointly produced academic articles to focus on the difference coproduction made - jointly submitted to the Health & Care Research Wales **national award for PPI** and won. - **Jointly produced a lay report** to share with multiple stakeholders including patients and family members. |


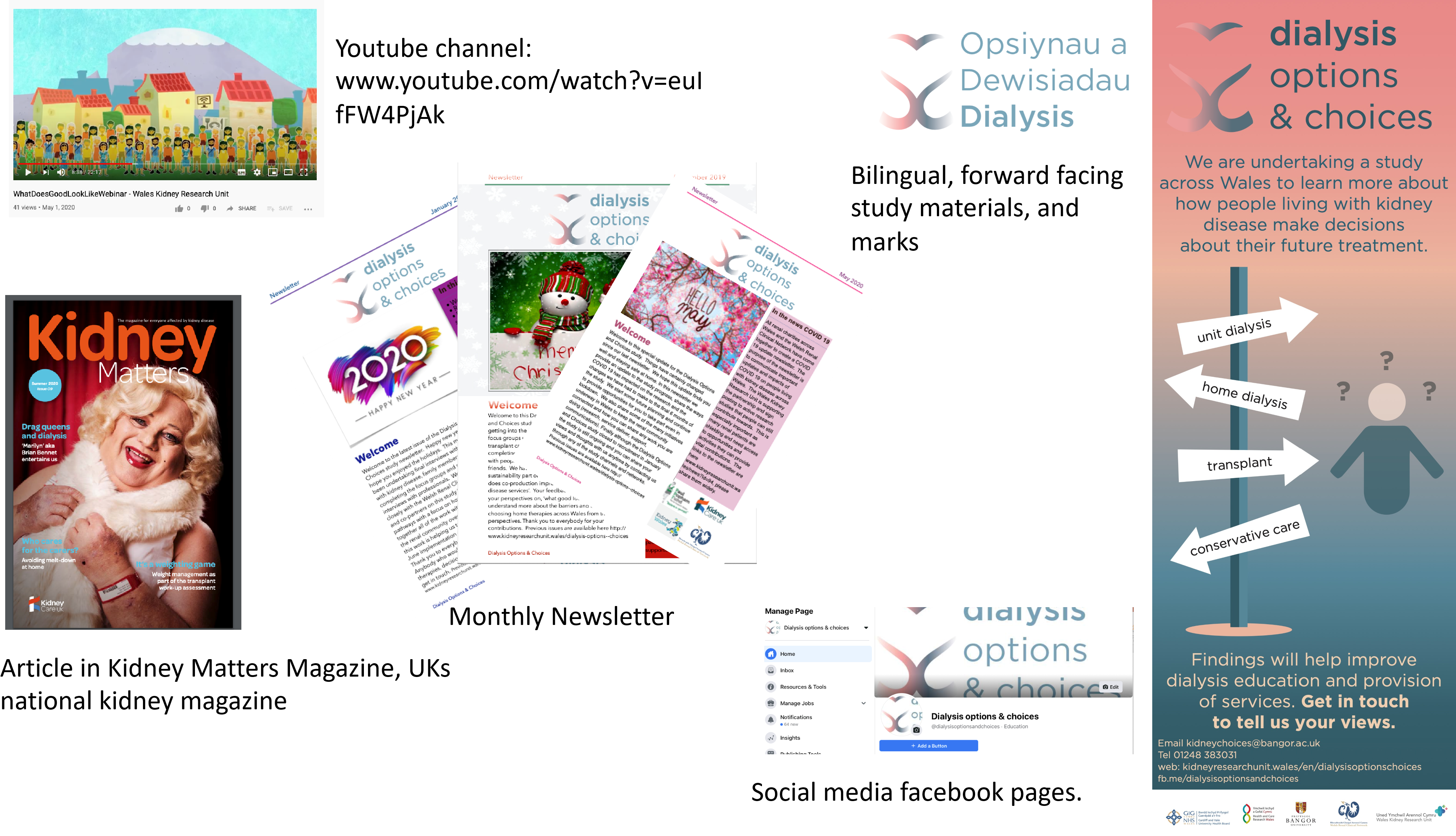
 Examples of the co-productive materials

GRIPP2 long form

| Section and topic | Item | Reported on page No |
| --- | --- | --- |
| Section 1: Abstract of paper | |  |
| 1a: Aim | Report the aim of the study | Page 1 |
| 1b: Methods | Describe the methods used by which patients and the public were involved | p. 1 |
| 1c: Results | Report the impacts and outcomes of PPI in the study | p.1 |
| 1d:Conclusions | Summarise the main conclusions of the study | p.1 |
| 1e: Keywords | Include PPI, “patient and public involvement,” or alternative terms as keywords | p.1 |
| Section 2: Background to paper | |  |
| 2a: Definition | Report the definition of PPI used in the study and how it links to comparable studies | p.4 |
| 2b: Theoretical underpinnings | Report the theoretical rationale and any theoretical influences relating to PPI in the study | p.4 |
| 2c: Concepts and theory development | Report any conceptual models or influences used in the study | p.5 |
| Section 3: Aims of paper | |  |
| 3: Aim | Report the aim of the study | p.6 |
| Section 4: Methods of paper | |  |
| 4a: Design | Provide a clear description of methods by which patients and the public were involved | p.6 |
| 4b: People involved | Provide a description of patients, carers, and the public involved with the PPI activity in the study | p.6 & table 1 supplementalfile7. |
| 4c: Stages of involvement | Report on how PPI is used at different stages of the study | p.6 Table 1, supplementalfile7. |
| 4d: Level or nature of involvement | Report the level or nature of PPI used at various stages of the study | Table 1, supplementalfile7. |
| Section 5: Capture or measurement of PPI impact | |  |
| 5a: Qualitative evidence of impact | If applicable, report the methods used to qualitatively explore the impact of PPI in the study | n/a |
| 5b: Quantitative evidence of impact | If applicable, report the methods used to quantitatively measure or assess the impact of PPI | n/a |
| 5c: Robustness of measure | If applicable, report the rigour of the method used to capture or measure the impact of PPI | n/a |
| Section 6: Economic assessment | |  |
| 6: Economic assessment | If applicable, report the method used for an economic assessment of PPI | n/a |
| Section 7: Study results | |  |
| 7a: Outcomes of PPI | Report the results of PPI in the study, including both positive and negative outcomes | p.7-9 table 2, fig.2. |
| 7b: Impacts of PPI | Report the positive and negative impacts that PPI has had on the research, the individuals involved (including patients and researchers), and wider impacts | p.7-9 table 2, fig.2. |
| 7c: Context of PPI | Report the influence of any contextual factors that enabled or hindered the process or impact of PPI | p.7-9 table 2, fig.2. |
| 7d: Process of PPI | Report the influence of any process factors, that enabled or hindered the impact of PPI | p.7-9 table 2, fig.2. |
| 7ei: Theory development | Report any conceptual or theoretical development in PPI that have emerged | p.10 |
| 7eii: Theory development | Report evaluation of theoretical models, if any | n/a |
| 7f: Measurement | If applicable, report all aspects of instrument development and testing (eg, validity, reliability, feasibility, acceptability, responsiveness, interpretability, appropriateness, precision) | n/a |
| 7g: Economic assessment | Report any information on the costs or benefit of PPI | p.8 |
| Section 8: Discussion and conclusions | |  |
| 8a: Outcomes | Comment on how PPI influenced the study overall. Describe positive and negative effects | p.10 |
| 8b: Impacts | Comment on the different impacts of PPI identified in this study and how they contribute to new knowledge | p.10 |
| 8c: Definition | Comment on the definition of PPI used (reported in the Background section) and whether or not you would suggest any changes | p.11 |
| 8d: Theoretical underpinnings | Comment on any way your study adds to the theoretical development of PPI | p.10 |
| 8e: Context | Comment on how context factors influenced PPI in the study | p.10 |
| 8f: Process | Comment on how process factors influenced PPI in the study | p.10 |
| 8g: Measurement and capture of PPI impact | If applicable, comment on how well PPI impact was evaluated or measured in the study | n/a |
| 8h: Economic assessment | If applicable, discuss any aspects of the economic cost or benefit of PPI, particularly any suggestions for future economic modelling. | p.10 |
| 8i: Reflections/critical perspective | Comment critically on the study, reflecting on the things that went well and those that did not, so that others can learn from this study | p.11 |

PPI=patient and public involvement reporting standards^80^
