# Supplementary material for: Assessing the efficacy of coproduction to better understand the barriers to achieving sustainability in NHS chronic kidney services and create alternate pathways
Source: Health Expect. 2021 Dec 28;25(2):579–606. doi: 10.1111/hex.13391 (PMC8957730; doi:10.1111/hex.13391)
Supplement: Supplementary file 3 — Supporting information. [file HEX-25--s003.docx]

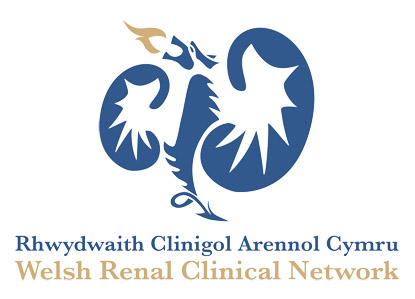


**Home Therapies Data – All Wales**

**April 2020**

This report contains data collected from VitalData correct for 30^th^ April 2020. The Home HD (HHD) activity data is collected at a 2 month time delay to allow time for data to be entered i.e. This report includes HHD activity data for up to and including February 2020.

It reports on the following key measures:

1. % of prevalent dialysis patients receiving haemodialysis treatment at home (HHD)
2. % of prevalent dialysis patients receiving peritoneal dialysis (PD) treatment
3. % of prevalent dialysis patients dialysing via a home therapy
4. Number of Home HD treatments recorded in VitalData

And compares the data with the following WRCN aspirational targets:

1. 15% of prevalent dialysis patients should be receiving haemodialysis (HD) treatment at home
2. 15% of prevalent dialysis patients should be receiving peritoneal dialysis (PD) treatment
3. 30% of prevalent dialysis patients should be receiving treatment via a home therapy

All measures are reported on for All Wales as well as for each of the three treatment regions.

Patient shortfalls for each treatment option in Tables 1-3 refer to the difference between the actual number of patients on each treatment and the number of patients required to meet the WRCN targets.

**Table 1:** April 2020 summary data for All Wales and each treatment health board

|  | | All Wales | 1 | 2 | 3 | WRCN target |
| --- | --- | --- | --- | --- | --- | --- |
| Number of prevalent dialysis patients | | 1486 | 361 | 618 | 507 |  |
|  | |  |  |  |  |  |
|  |  |  |  |  |  |  |
|  | |  |  |  |  |  |
| Number of dialysis patients receiving a home therapy treatment | | 292 | 80 | 93 | 119 |  |
| % of dialysis patients receiving a home therapy treatment | | 19.7 | 22.2 | 15.0 | 23.5 | 30 |
| Total number of patients required to meet 30% target | | 446 | 109 | 186 | 153 |  |
| Current patient shortfall | | -154 | -29 | -93 | -34 |  |
|  | |  |  |  |  |  |
| Number of dialysis patients receiving unit HD treatment | | 1194 | 281 | 525 | 388 |  |
| % of dialysis patients receiving unit HD treatment | | 80.3 | 77.8 | 85.0 | 76.5 |  |

*1 PD patient in North Wales had ‘PD type unknown’*

**Table 2:** April 2020 summary data for All Wales and each treatment centre

|  | | All Wales | 1 | 2 | 3 | 4 | 5 | WRCN target |
| --- | --- | --- | --- | --- | --- | --- | --- | --- |
| Number of prevalent dialysis patients | | 1486 | 103 | 109 | 149 | 618 | 507 |  |
|  |  |  |  |  |  |  |  |  |
|  | |  |  |  |  |  |  |  |
| Number of dialysis patients receiving a home therapy treatment | | 292 | 32 | 18 | 30 | 93 | 119 |  |
| % of dialysis patients receiving a home therapy treatment | | 19.7 | 31.1 | 16.5 | 20.1 | 15.0 | 23.5 | 30 |
| Total number of patients required to meet 30% target | | 446 | 31 | 33 | 45 | 186 | 153 |  |
| Current patient shortfall | | -154 | 0 | -15 | -15 | -93 | -34 |  |
|  | |  |  |  |  |  |  |  |
| Number of dialysis patients receiving unit HD treatment | | 1194 | 71 | 91 | 119 | 525 | 388 |  |
| % of dialysis patients receiving unit HD treatment | | 80.3 | 68.9 | 83.5 | 79.9 | 85.0 | 76.5 |  |

*1 PD patient in 1 had ‘PD type unknown*
